# Localization on a-priori information of plane extraction

**DOI:** 10.1371/journal.pone.0285509

**Published:** 2023-05-08

**Authors:** Junjie Ji, Jing-Shan Zhao, Sergey Yurievich Misyurin, Daniel Martins

**Affiliations:** 1 Department of Mechanical Engineering, Tsinghua University, Beijing, China; 2 Moscow Engineering Physics Institute, National Research Nuclear University MEPhI, Moscow, Russia; 3 Blagonravov Mechanical Engineering Research Institute RAS, Moscow, Russia; 4 Department of Mechanical Engineering, Federal University of Santa Catarina, Florianópolis, Brazil; Kocaeli University, TURKEY

## Abstract

Localization constitutes a critical challenge for autonomous mobile robots, with flattened walls serving as a fundamental reference for indoor localization. In numerous scenarios, prior knowledge of a wall’s surface plane is available, such as planes in building information modeling (BIM) systems. This article presents a localization technique based on a-priori plane point cloud extraction. The position and pose of the mobile robot are estimated through real-time multi-plane constraints. An extended image coordinate system is proposed to represent any planes in space and establish correspondences between visible planes and those in the world coordinate system. Potentially visible points representing the constrained plane in the real-time point cloud are filtered using the filter region of interest (ROI), derived from the theoretical visible plane region within the extended image coordinate system. The number of points representing the plane influences the calculation weight in the multi-plane localization approach. Experimental validation of the proposed localization method demonstrates its allowance for redundancy in initial position and pose error.

## 1. Introduction

The localization problems are of paramount importance for mobile robotics, particularly for indoor robots operating in environments where external navigational aids such as GPS prove to be unreliable or unavailable. For example, painting robots localize themselves to apply paint on target surfaces within multiple rooms [1, 2], while inspecting power distribution cabinets necessitates localization to identify the intended targets [3]. Furthermore, localization serves as an integral component of simultaneous localization and mapping (SLAM), a widely employed technique for robotic navigation in uncharted environments [4–6].

The challenges associated with mobile robot localization are heightened in settings where distinctive features are either sparse or repetitive, such as indoor construction sites [7, 8]. In these scenarios, feature-based localization methods face significant challenges due to the lack of unique and easily identifiable features.

In contrast, indoor construction sites often possess structured information in the form of blueprints such as Building Information Model (BIM), which detail the locations of walls and other architectural elements [9]. This a-priori knowledge presents an opportunity to enhance conventional feature-based localization methods by integrating the structured parameters into the localization procedure.

The development of innovative localization techniques that can effectively utilize structured information from blueprints, has emerged as a challenge for robotics developers. These methods aim to augment the localization performance of mobile robots in indoor construction sites and other environments lacking distinct features, yet possessing adequate plane characteristics for localization purposes.

### 1.1 Review of related works

Solving the localization problem is a prerequisite for mobile robots to perform tasks effectively. Robots can utilize various methods for localization, such as magnetic [10] or sonar-based techniques [11]. Integrating historical signals and fusing data from multiple sensors are also crucial to enhance localization accuracy and robustness [12]. As research progresses, innovative approaches will continue to emerge, addressing diverse localization scenarios and advancing mobile robot capabilities.

The utilization of visual information for mobile robot localization is a crucial aspect of navigation, and it has been the subject of extensive research. Two primary categories of methods have emerged, including direct [13, 14] and indirect methods [15]. Direct methods involve the analysis of raw sensor data, such as depth [16, 17] or pixel information [18], to estimate the robot’s position and orientation within its environment. Some direct methods leverage the initial position and pose estimates to enhance localization performance [19]. Conversely, indirect methods concentrate on extracting distinct features from the environment and employing these features for localization purposes [20]. Indirect methods of localization typically involve extracting features from the sensor data, which are subsequently utilized to estimate the robot’s pose [21]. Feature-based methods can offer greater efficiency compared to direct methods, as they operate on a diminished set of data points [22–24]. However, the efficacy of indirect methods can be influenced by the quality and uniqueness of the extracted features, which might perform difficult to acquire in environments characterized by featureless planes or repetitive structures.

A widely employed primitive in indoor environments is the plane primitive, which is frequently utilized in the localization process. Furthermore, planes typically serve as the basis for measurements [25, 26]. A pi-SLAM [27] introduced a real-time dense planar LiDAR-based localization system that employs planes as landmarks. Zhou *et al*. [28] outperformed localization methods using planes, lines, and cylinders. Wen *et al*. [29] utilized the absolute ground plane to constrain vertical pose estimation, subsequently reducing the estimation error. A point-plane-object localization system was proposed for semantic map reconstruction [30], which demonstrated effective localization in indoor scenarios. However, the method in [30] necessitates strict criteria, such as planes being parallel or perpendicular to one another. Additionally, in both [27, 28], planes are identified solely by analyzing point clouds. Moreover, the extracted planes, represented by point clouds, demand an extra registration process to incorporate the planes into the existing map. In low-texture environments, robots can leverage intersection lines between walls and floors to model walls and scenes [31]. However, this method requires that the intersection lines be visible. Region-of-interest (ROI) approaches are well-suited for extracting relevant point clouds [32, 33]. When planar surfaces within a scene lack notable distinctions or exhibit visual similarity, a priori (ROI) information can be employed to segment and differentiate these planes [34]. The segmented planes facilitate matching the observed planes with structured information from blueprints or other sources, thereby improving localization performance.

Building Information Modeling (BIM) technology enhances the informativeness, intelligence, and eco-friendliness of the construction industry [35]. Numerous studies have explored the integration of a-priori BIM blueprint data with mobile robot localization [1, 9, 36]. The a-priori plane information of a construction site is readily accessible from the digital model [37]. The a priori known planes from BIM models boast extensive applicability. These planes are extracted to facilitate the detection, identification, and localization of lighting elements [38]. Autonomous monitoring of construction progress is achieved by comparing BIM models to the actual, acquired photogrammetric point clouds [39]. Zhao *et al*. [9] proposed a feature-based method to localize mobile robots by matching features between online information and the geometric and semantic information retrieved from BIM. However, this method is constrained by feature qualities and is ill-suited for featureless scenarios. Schaub *et al*. [36] localized mobile robots by matching the entire point cloud in the scene to the complete point cloud generated by BIM. Nonetheless, matching entire point clouds necessitates high-quality online point cloud observations. Drawing from these localization studies, it becomes apparent that utilizing only a portion of the online point cloud in a scene is sufficient for a mobile robot to determine its location. By combining this with the commonly present plane primitives in indoor environments, mobile robots can leverage multiple a-priori planes for localization.

### 1.2 Objectives and scope of this study

In light of the deficiencies inherent in existing localization methods, this paper proposes a novel localization approach that utilizes a-priori known planes in the absolute environment. The main idea of this method involves using the mobile robot’s coarse initial pose and the absolute position of planes in the map to extract planes from the online-obtained point cloud via the region of interest (ROI) method. Subsequently, by comparing plane poses representedin the camera coordinate system and ground coordinate system, the refined pose and position of the robot can be determined. This method does not require the visibility of intersection lines and vertices of the planes.

The main contributions of this paper include:

- An approach that employs a-priori absolute reference planes and the mobile robot’s coarse initial pose to extract plane point clouds from online-obtained point cloud.
- An extended image coordinate system that aids in locating invisible primitives and determining the visibility of any reference plane.
- A multi-plane localization algorithm that calculates the robot’s pose and position using the a priori planes’ absolute poses and the observed planes within view.

The remainder of the paper is organized as follows. Section 2 presents the problem formulation of localization with multiple planes. Section 3 outlines the method details of localization, with Section 3.1 introducing the method of representing arbitrary planes in the extended image system to help determine whether reference planes are visible. Section 3.2 proposes the algorithm for localization with multiple planes, and Section 3.3 provides an overview of the proposed methods for localization. Section 4 discusses experimental tests on algorithm feasibility. Lastly, Section 5 and Section 6 present the discussion and conclusion, respectively.

## 2 problem formulation

Suppose a world coordinate system *O*_W_*x*_W_*y*_W_*z*_W_, exists. A mobile robot’s fundamental characteristic is its motion along the ground. As the robot’s altitude remains constant while moving, the ground coordinate system, *O*_G_*x*_G_*y*_G_*z*_G_, is established. When the ground surface is flat, the mobile robot moves within the *x*_G_*Oy*_G_ plane of the ground coordinate system *O*_G_*x*_G_*y*_G_*z*_G_. If the coordinates, *P*_r_(*x*,*y*,*z*), depict the robot’s position, the *z*-axis component is zero.

When considering movement between floors or uneven terrain, the ground coordinate system, *O*_G_*x*_G_*y*_G_*z*_G_, does not coincide with the world coordinate system *O*_W_*x*_W_*y*_W_*z*_W_. The homogeneous transformation matrix from the ground coordinate system, *O*_G_*x*_G_*y*_G_*z*_G_, to the world coordinate system, *O*_W_*x*_W_*y*_W_*z*_W_, is defined as $T_{G}^{W}$. The ground coordinate system serves as the primary reference for the robot’s motion. In this paper, the ground coordinate system is assumed to be unique. If multiple ground coordinate systems exist due to uneven terrain, the proposed method remains applicable by replacing the transformation matrix ${\left(T_{G}^{W}\right)}_{i}$.

A robot coordinate system, *O*_R_*x*_R_*y*_R_*z*_R_, and the corresponding homogeneous transformation matrix, $T_{R}^{G}$, are defined to easily express surrounding objects’ relative positions to the mobile robot. The robot is situated at the origin of the robot coordinate system. The *z*_*R*_ axis of *O*_R_*x*_R_*y*_R_*z*_R_ is parallel to the *z*_G_ axis of *O*_G_*x*_G_*y*_G_*z*_G_, while the *x*_R_*Oy*_R_ plane of *O*_R_*x*_R_*y*_R_*z*_R_ coincides with the *x*_G_*Oy*_G_ of *O*_G_*x*_G_*y*_G_*z*_G_.

**Fig 1:**
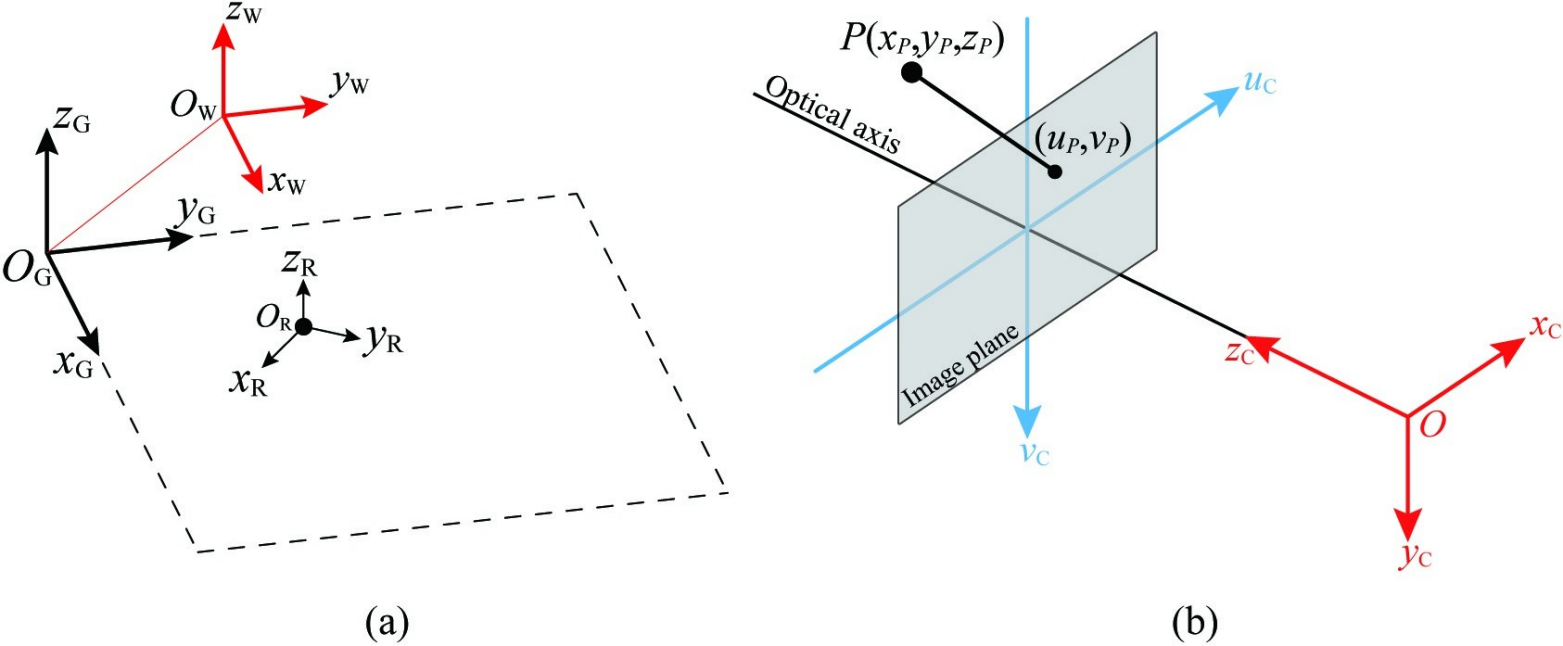
Relevant coordinate systems. (a)The relative spatial relationship between the world coordinate system, the ground coordinate system, and the robot coordinate system. (b) The camera coordinate system and the image coordinate system.

The relative spatial relationships of the aforementioned coordinate systems are illustrated in Fig 1(A).

As a mobile robot moves on the ground, its pose and position can be represented by three parameters, *x*, *y* and *φ*. The parameters *x* and *y* denote the mobile robot’s coordinates on the ground plane *x*_G_*Oy*_G_. The parameter *φ* represents the mobile robot’s pose angle, with the quantitative value defined as the included angle between the robot’s forward direction and the *x*-axis direction vector of the ground coordinate system *O*_G_*x*_G_*y*_G_*z*_G_.

The homogeneous transformation matrix, $T_{R}^{G}$, can be expressed by pose parameters (*x*,*y*,*φ*), as shown in Eq (1).


$$T_{R}^{G}=[\begin{array}{cccc} \cos\varphi & \sin\varphi & 0 & x\\ -\sin\varphi & \cos\varphi & 0 & y\\ 0 & 0 & 1 & 0\\ 0 & 0 & 0 & 1 \end{array}]$$ (1)

Suppose the homogenous coordinates in the robot coordinate system are ***p***^R^, and the corresponding homogenous coordinates of *P*^R^ in the ground coordinate system are ***p***^G^. The transformation equation is illustrated in Eq (2).

$$p^{G}=T_{R}^{G}p^{R}$$ (2)

where the transformation matrix $T_{R}^{G}$ is defined by Eq (1).

Cameras are fixed on the mobile robot. The camera coordinate system is a 3-D coordinate system established with the camera placed at the origin. The conventional image coordinate system is a 2-D coordinate system, representing the situation the image locates. Fig 1(b) shows the arrangement of camera coordinate system and the image coordinate system.

There is a point in the camera coordinate system, *P*(*x*_*P*_,*y*_*P*_,*z*_*P*_,1), and its corresponding coordinates in the conventional image coordinate system, *P*(*u*_*P*_,*v*_*P*_,1). Above coordinates are represented in homogenous coordinates. The transformation equation from the camera coordinates to the conventional image coordinates can be expressed as follow

$$z_{P}[\begin{array}{c} u_{P}\\ v_{P}\\ 1 \end{array}]=[\begin{array}{cccc} f_{x} & 0 & u_{0} & 0\\ 0 & f_{y} & v_{0} & 0\\ 0 & 0 & 1 & 0 \end{array}][\begin{array}{c} x_{P}\\ y_{P}\\ z_{P}\\ 1 \end{array}]$$ (3)

where *f*_*x*_,*f*_*y*_,*u*_0_,*v*_0_ represents the internal parameters of the camera.

Assume that the homogeneous representation of the plane, *π*, is defined as Eq (4).

$$\pi ={\left(\pi_{1},\pi_{2},\pi_{3},\pi_{4}\right)}^{T}$$ (4)

where the elements, *π*_1_,*π*_2_,*π*_3_,*π*_4_, separately represent the coefficients of the plane *π*. The generalized Equation of a plane can be expressed using the homogeneous representation, as shown in Eq (5).


$$\pi_{1}x+\pi_{2}y+\pi_{3}z+\pi_{4}=0$$ (5)

Consider a plane *π*_*i*_ with its representation in the ground coordinate system as $\pi_{i}^{G}$. The plane is directly observed by the camera, and its representation in the camera coordinate system is *π*^C^. The transformation equation of the two-plane representations is shown in Eq (6).

$$\pi_{i}^{G}={\left(T_{R}^{G}\right)}^{-T}{\left(T_{C}^{R}\right)}^{-T}\pi_{i}^{C}$$ (6)

where $T_{R}^{G}$ represents the transformation matrix from the robot coordinate system to the ground coordinate system, and $T_{C}^{R}$ represents the transformation matrix from the camera coordinate system to the robot coordinate system. The derivation of Eq (6) can be found in S1 Appendix.

The matrix $T_{R}^{G}$ denotes the homogeneous transformation matrix from the robot coordinate system to the ground coordinate system. Its structure is determined by the pose vector, (*x*,*y*,*φ*), according to Eq (1).

The matrix $T_{C}^{R}$ denotes the homogeneous transformation matrix from the camera coordinate system to the robot coordinate system. Its structure is determined by the mechanical connection geometry between the camera and the robot. When the camera is mounted on the mobile robot, the matrix $T_{C}^{R}$ is considered invariant.

Since Eq (6) is established by the vision system’s observation, and the pose vector serves as the unknown variables, it describes a position and pose constraint for the mobile robot. Multiple constraints in the form of Eq (6) constitute a constraint equation system. The position and pose of the mobile robot are estimated by solving this constraint equation system.

In Eq (7), multiple planes are observed simultaneously by the camera system, and the constraint equation system can be expressed as follows.


$$S=\{(x,y,\varphi )|{\left(\pi_{j}^{i}\right)}^{G}={\left(T_{R}^{G}(x,y,\varphi )\right)}^{-T}{\left(T_{C\_i}^{R}\right)}^{-T}{\left(\pi_{j}^{i}\right)}^{C}\}$$ (7)

In Eq (7), ***S*** represents the constraint equation system, (*x*,*y*,*φ*) represents the position and pose estimation from the constraint equation system. The superscript *i* of $\pi_{j}^{i}$ represents the sequence of the camera, where 1≤*i*≤*n*, and *n* denotes the number of the cameras in the vision system. The subscript *j* of $\pi_{j}^{i}$ represents the plane sequence that exist in the view field of camera *i*, where 1≤*j*≤*m*_*i*_, and *m*_*i*_ denotes the number of the planes exist in view field of camera *i*. ${\left(\pi_{j}^{i}\right)}^{G}$ represents the plane $\pi_{j}^{i}$ expressed in the ground coordinate system. ${\left(\pi_{j}^{i}\right)}^{C}$ represents the plane $\pi_{j}^{i}$ expressed in the camera coordinate system. $\left(T_{R}^{G}(x,y,\varphi )\right)$ represents the homogenous transformation matrix from robot coordinate system to the ground coordinate system. $T_{C\_i}^{R}$ denotes the homogenous transformation matrix from the camera *i* to the robot coordinate system.

According to the constraint equation system shown in Eq (7), a plane, $\pi_{j}^{i}$, might be observed by more than one camera at the same time. In other words, the plane $\pi_{j}^{i}$ with different identification parameters, *i*, *j*, may represent the same plane in a real-world environment. However, the same plane observed by different cameras provides different constraints. These additional constraints help to generate more precise estimations of position and pose.

Two main challenges arise when solving the constraint equation system. The first is how to establish the accurate connections between real-time observed planes and a-priori known plane parameters. The second is how to find the optimal solution for the constraint equation system. The remainder of this article will discuss these two problems separately.

## 3 Establishing and solving constraint equations with a-priori information

### 3.1 Overview of proposed method

In a scene with multiple planes that lack distinctive features, the primary challenge when localizing a mobile robot lies in identifying and distinguishing these planes within the current field of view. To address this issue, the proposed method for localizing mobile robots based on a-priori map information combines the robot’s current coarse pose estimate with the map’s a-priori information. This enables the position estimation of the planes used for localization within the field of view, allowing for the matching of observed planes with their corresponding a-priori map planes. Each camera’s online identification of a plane and its mapping relationship in the map constitute a pose constraint for the mobile robot.

When the number of planes in the field of view is redundant, an optimal estimation of the robot’s pose is achieved through a weighted least squares approach. This approach takes into account the uncertainty of plane identification as the weight and ensures accurate and robust pose estimation.

**Fig 2:**
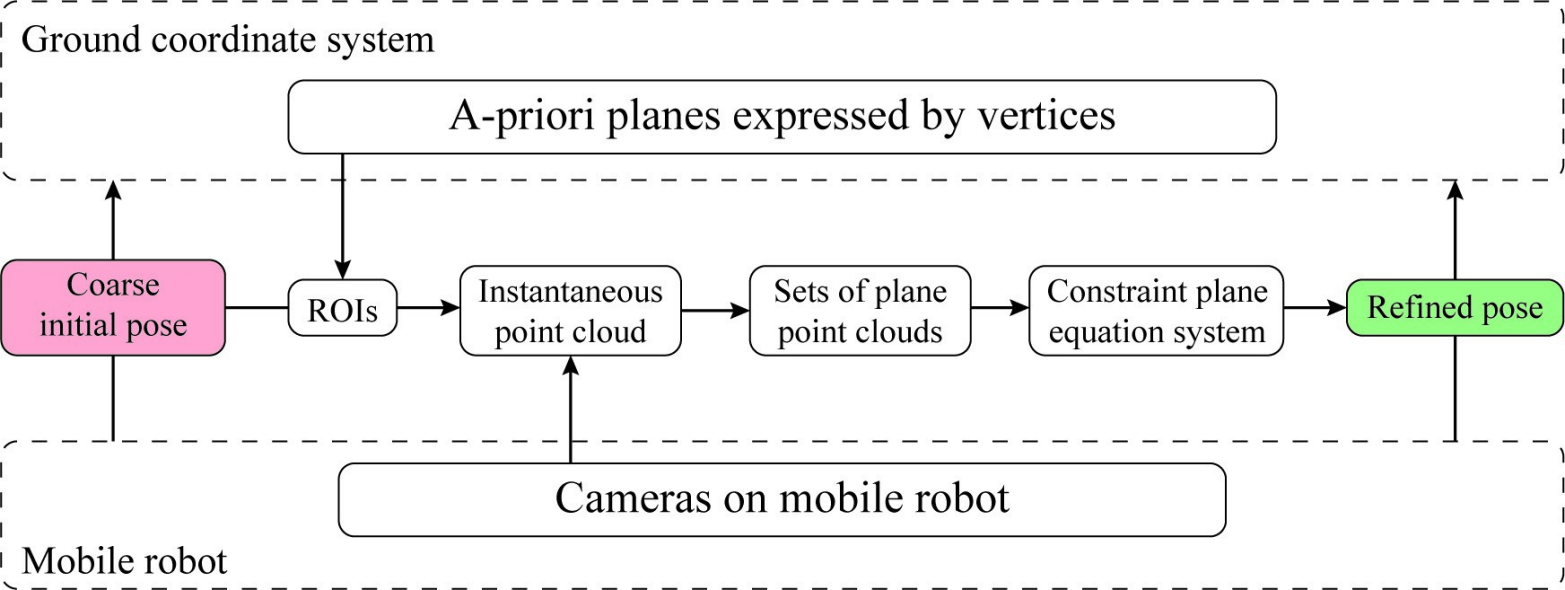
Processing flow chart of the proposed method.

The flowchart of the proposed method is shown in Fig 2.

As shown in Fig 2, the a-priori planes expressed by their vertices represent the information of the a-priori known map, all of which are represented in the ground coordinate system. The a-priori planes can be obtained from the building blueprint in BIM or from the previous map constitution. The coarse transformation matrix between the camera coordinate system and the ground coordinate system is known according to the coarse initial pose. An extended image coordinate system is set up to express invisible points in the camera coordinate system. The ROIs corresponding to each theoretically visible plane are determined using the coarse initial pose and a-priori planes.

Using the obtained ROIs and the instantaneous point cloud online captured by the cameras, the entire point cloud is segmented into each plane suitable for localization. After segmentation using the ROIs, the parameters of each plane are identified by the random sample consensus (RANSAC) method. These identified parameters form the constraint plane equation system, where the unknown variables of the equation system represent the position and pose.

The constraint equation system is solved to estimate the mobile robot’s position and pose. If the size of the equation system is more than two, a weighted least squares method is used to obtain the optimal estimation, ensuring accurate and robust pose estimation for the mobile robot.

### 3.2 Identifying localization reference planes using the extended image coordinate system

In conventional image plane coordinate systems, object representation is confined to the field of view. To enable the depiction of arbitrary spatial objects and conveniently ascertain a plane’s visibility within the field of view, an extended image coordinate system has been devised.

Consider a spatial point, *P*(*x*,*y*,*z*), within the camera coordinate system. The components of coordinates in the image coordinates are depicted in Eq (8) (*x*≠0,*y*≠0).

$$\left\{ \begin{array}{l} u=\frac{2x}{\theta_{h}|x|}\arccos\frac{z}{\sqrt{x^{2}+z^{2}}}\\ v=\frac{2y}{\theta_{v}|y|}\arccos\frac{z}{\sqrt{y^{2}+z^{2}}} \end{array}\right.$$ (8)

where *θ*_h_ and *θ*_v_ signifying the camera’s horizontal and vertical field of view, respectively, and (*u*,*v*) denoting the coordinates within the extended image coordinates system.

If *x* = 0,*y*≠0, Eq (8) is supplanted by Eq (9).


$$\left\{ \begin{array}{l} u=\frac{2z}{\theta_{h}|z|}\arccos\frac{z}{\left|z\right|}\\ v=\frac{2y}{\theta_{v}|y|}\arccos\frac{z}{\sqrt{y^{2}+z^{2}}} \end{array}\right.$$ (9)

And if *x*≠0,*y* = 0, Eq (8) is supplanted by (10).


$$\left\{ \begin{array}{l} u=\frac{2x}{\theta_{h}|x|}\arccos\frac{z}{\sqrt{x^{2}+z^{2}}}\\ v=\frac{2z}{\theta_{v}|z|}\arccos\frac{z}{\left|z\right|} \end{array}\right.$$ (10)

When *x* = 0,*y*≠0, if *z*<0, Eq (8) is supplanted by (11)

$$\left\{ \begin{array}{c} u=0\\ v=0 \end{array}\right.$$ (11)

If *z*<0, Eq (8) is supplanted by (12)

$$\left\{ \begin{array}{l} u=-\frac{2\pi }{\theta_{h}}\\ v=-\frac{2\pi }{\theta_{v}} \end{array}\right.$$ (12)

The value domain of all points within the camera coordinate system after transformation is expressed in Eq (13).


$$\left\{ \begin{array}{l} u\in \left[-\frac{2\pi }{\theta_{h}},\frac{2\pi }{\theta_{h}}\right)\\ v\in \left[-\frac{2\pi }{\theta_{v}},\frac{2\pi }{\theta_{v}}\right) \end{array}\right.$$ (13)

**Fig 3:**
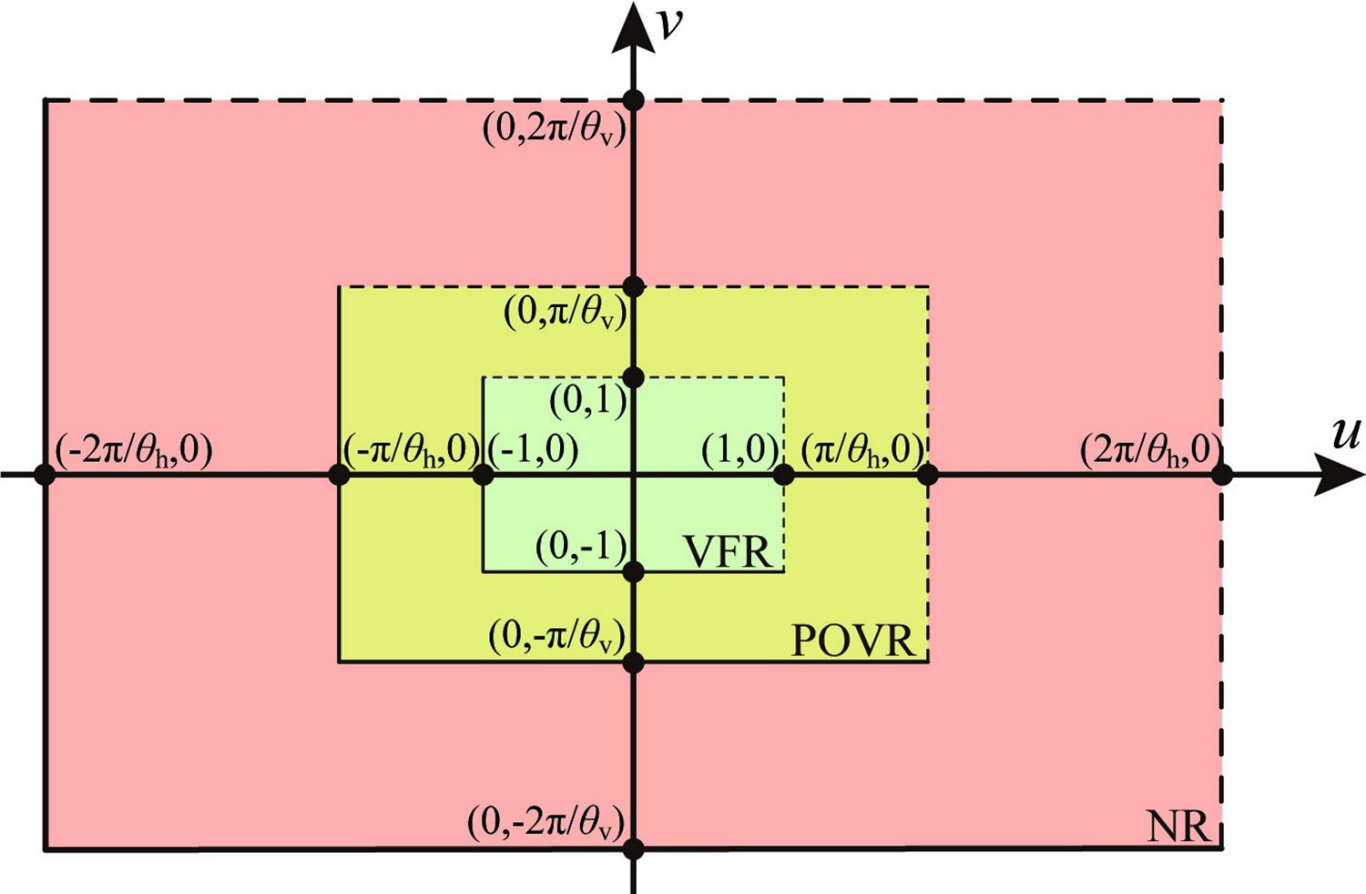
Extended image coordinate system and sub-regions.

Upon mapping each point from the camera coordinate system to the extended image coordinate system, the comprehensive extended coordinate system comprises three regions, as illustrated in Fig 3.

In the extended image coordinate system, Fig 3 demonstrates that the view field region (VFR), colored green, is defined by *u*∈[−1,1) and *v*∈[−1,1). The positive out-of-view field region (POVR), colored yellow, is defined by *u*∈[−π/*θ*_h_,π/*θ*_h_) and *v*∈[−π/*θ*_v_,π/*θ*_v_) outside the VFR. And the negative region (NR), colored red, is defined by *u*∈[−2π/*θ*_h_,2π/*θ*_h_) and *v*∈[−2π/*θ*_v_,2π/*θ*_v_).

**Fig 4:**
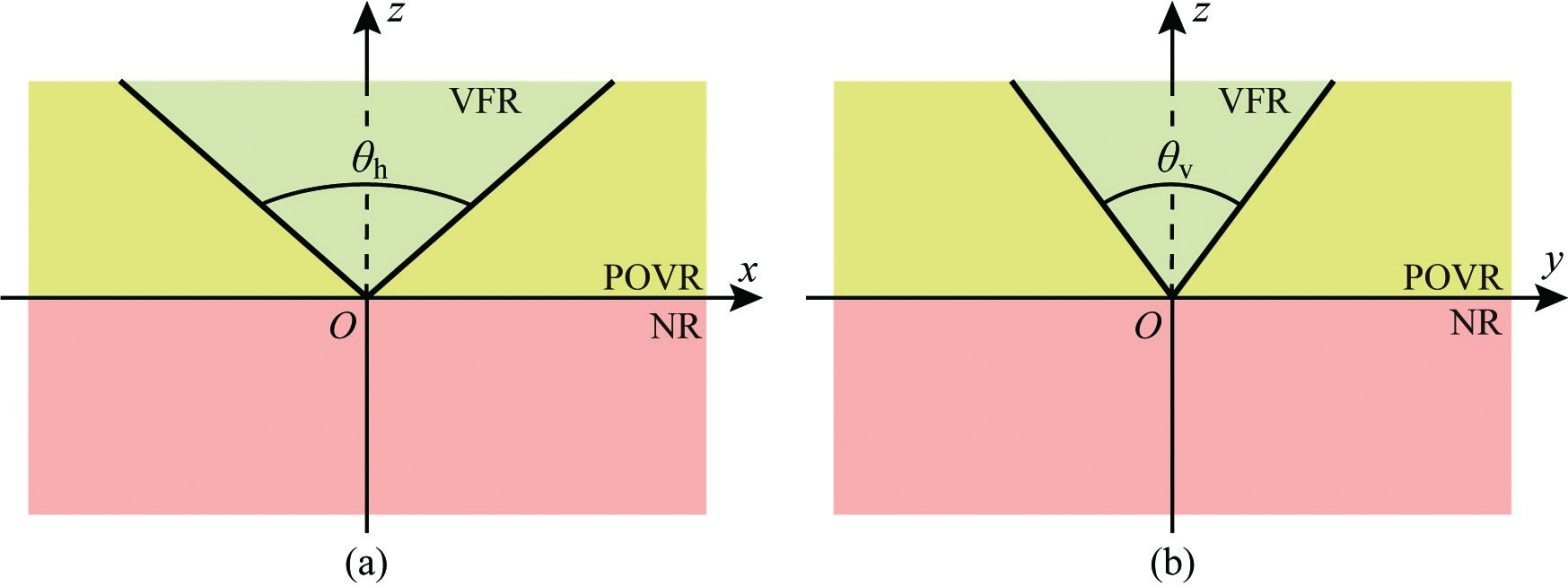
Subregions of camera coordinate system. (a) *xOz* plane view. (b) *yOz* plane view.

These three regions in the extended image coordinate system correspond to those in the camera coordinate system. Fig 4(A) and 4(B) depict the distribution of these regions from the planes *y* = 0 and *x* = 0, respectively.

**Fig 5:**
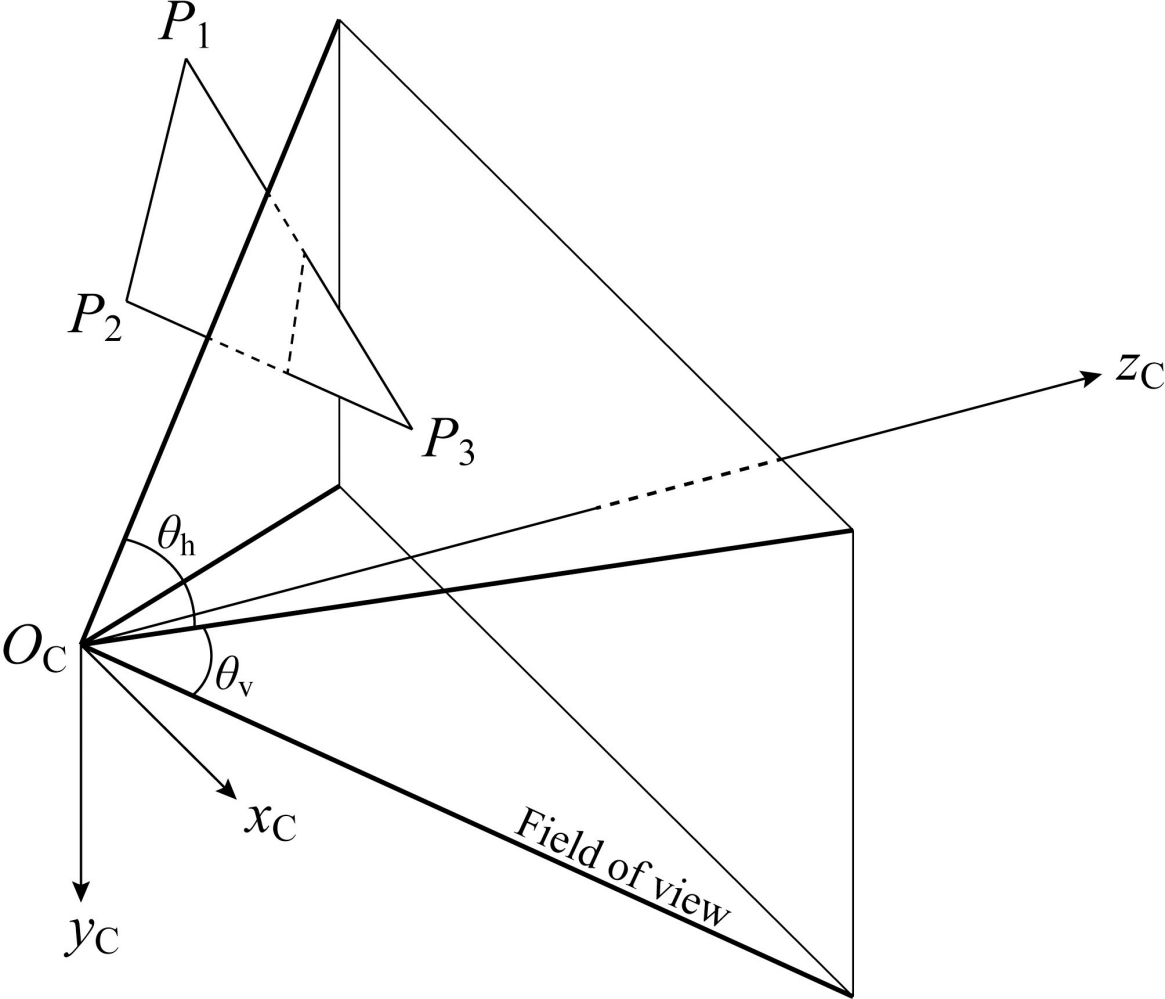
Relationship between plane primitive and the field of view.

A point within the VFR of the extended image coordinate system is visible. However, a plane’s visibility does not necessitate the visibility of its vertices. The most basic unit of the plane primitive is the triangle, exemplified by vertices *P*_1_(*x*_1_,*y*_1_,*z*_1_), *P*_2_(*x*_2_,*y*_2_,*z*_2_), and *P*_3_(*x*_3_,*y*_3_,*z*_3_), which represent a-priori known plane for localization, as displayed in Fig 5. The reference plane vertices’ coordinates are calculated based on the robot’s coarse initial pose and position. Each triangle vertex must reside within one of the extended image coordinate system’s regions.

Fig 5 demonstrates that as long as a portion of the a-priori plane is visible, the corresponding point cloud within the field of view can be extracted for localization. By assessing the closed shape vertices’ presence within the view region, the visibility issue can be partially resolved. Ten potential region distribution scenarios exist, and the objective of differentiating the triangle vertices’ area distribution within the extended image coordinate system is to expediently determine whether the bounded plane is partially situated within the camera field of view. The intersection status check table is presented in Table 1.

**Table 1:** Intersection status check table.

Seq.	Number in VR	Number in POVR	Number in NR	Intersect status
**1**	3	0	0	Intersect
**2**	2	0	1	Intersect
**3**	2	1	0	Intersect
**4**	1	0	2	Intersect
**5**	1	1	1	Intersect
**6**	1	2	0	Intersect
**7**	0	0	3	Unintersect
**8**	0	1	2	To be checked
**9**	0	2	1	To be checked
**10**	0	3	0	To be checked

Seven out of the ten possible distribution scenarios can be ascertained directly, while the remaining three necessitate further calculations to verify their visibility. The planes composing the view quadrangular pyramid, as shown in Fig 5, articulated by Eq (14).


$$\{\begin{array}{c} (\cos\frac{\theta_{h}}{2})x-(\sin\frac{\theta_{h}}{2})z=0\\ (\cos\frac{\theta_{h}}{2})x+(\sin\frac{\theta_{h}}{2})z=0\\ (\cos\frac{\theta_{v}}{2})y-(\sin\frac{\theta_{v}}{2})z=0\\ (\cos\frac{\theta_{v}}{2})y+(\sin\frac{\theta_{v}}{2})z=0 \end{array}$$ (14)

The prerequisite for the plane primitive *P*_1_*P*_2_*P*_3_ visibility is that one of the segments *P*_*i*_*P*_*j*_ intersects with any boundary plane, and the intersection point between the segments and the boundary plane should be situated at the VFR within the extended image coordinate system. The status check table aids in reducing computational expense. If the triangle employed for robot localization is absent from the field of view, further intersection calculations are rendered unnecessary.

### 3.3 Estimating position and pose from the constraint equation system

**Fig 6:**
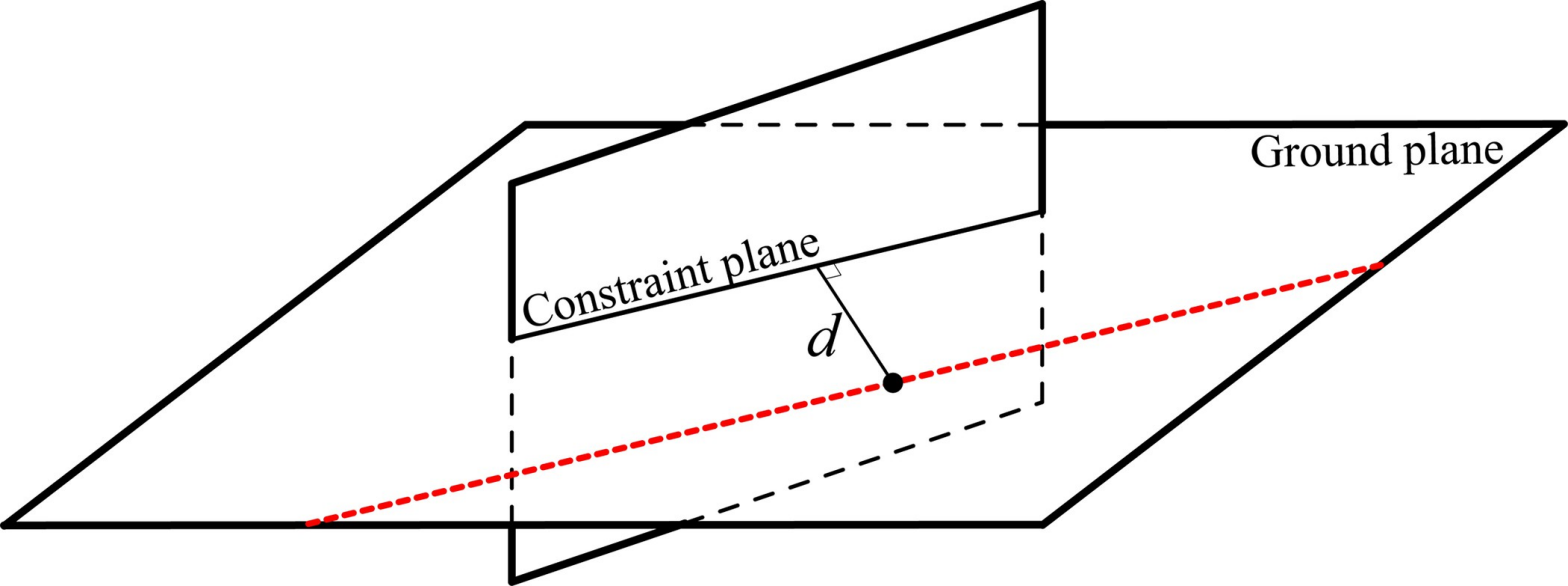
The plane constraint for the mobile robot.

Each visible plane restricts the mobile robot’s position and orientation to a line on the ground, with this constraint line being parallel to the intersection of the constraint plane and the ground. The constraint plane and the ground line are depicted in Fig 6.

As illustrated in Fig 6, the constraint plane intersects the ground, and the constraint line, parallel to the intersection line, is rendered in red. The plane equation, *π*^R^, represented in the robot coordinate system is conveyed in Eq (15).

$$\pi_{1}^{R}x+\pi_{2}^{R}y+\pi_{3}^{R}z+\pi_{4}^{R}=0$$ (15)

where $\pi_{1}^{R},\pi_{2}^{R},\pi_{3}^{R},\pi_{4}^{R}$ represent the coefficients of the plane *π*^R^. And the plane equation, *π*^G^, represented in the ground coordinate system is expressed in Eq (16)

$$\pi_{1}^{G}x+\pi_{2}^{G}y+\pi_{3}^{G}z+\pi_{4}^{G}=0$$ (16)

where $\pi_{1}^{G},\pi_{2}^{G},\pi_{3}^{G},\pi_{4}^{G}$ represent the coefficients of the plane *π*^G^.

The distance between the mobile robot’s center and the plane within both coordinate systems remains constant. In the ground coordinate system, this distance is expressed in Eq (17).

$$d_{G}=\frac{\left|\pi_{1}^{G}x+\pi_{2}^{G}y+\pi_{4}^{G}\right|}{\sqrt{{\left(\pi_{1}^{G}\right)}^{2}+{\left(\pi_{2}^{G}\right)}^{2}+{\left(\pi_{3}^{G}\right)}^{2}}}$$ (17)

where *d*_G_ symbolizes the distance between the target plane and mobile robot’s center.

In the robot coordinate system, this distance is conveyed in Eq (18)

$$d_{R}=\frac{\left|\pi_{4}^{R}\right|}{\sqrt{{\left(\pi_{1}^{R}\right)}^{2}+{\left(\pi_{2}^{R}\right)}^{2}+{\left(\pi_{3}^{R}\right)}^{2}}}$$ (18)

where *d*_R_ represents the distance between the target plane and mobile robot’s center.

Given that the distance invariance illustrated in Eq (19).


$$d_{G}=d_{R}$$ (19)

Substituting Eq (17), Eq (18) into Eq (19), yields Eq (20).


$$\left|\pi_{1}^{G}x+\pi_{2}^{G}y+\pi_{4}^{G}|\frac{\sqrt{{\left(\pi_{1}^{R}\right)}^{2}+{\left(\pi_{2}^{R}\right)}^{2}+{\left(\pi_{3}^{R}\right)}^{2}}}{\sqrt{{\left(\pi_{1}^{G}\right)}^{2}+{\left(\pi_{2}^{G}\right)}^{2}+{\left(\pi_{3}^{G}\right)}^{2}}}=|\pi_{4}^{R}\right|$$ (20)

Eq (20) represents the position constraint for the mobile robot on the ground.

**Fig 7:**
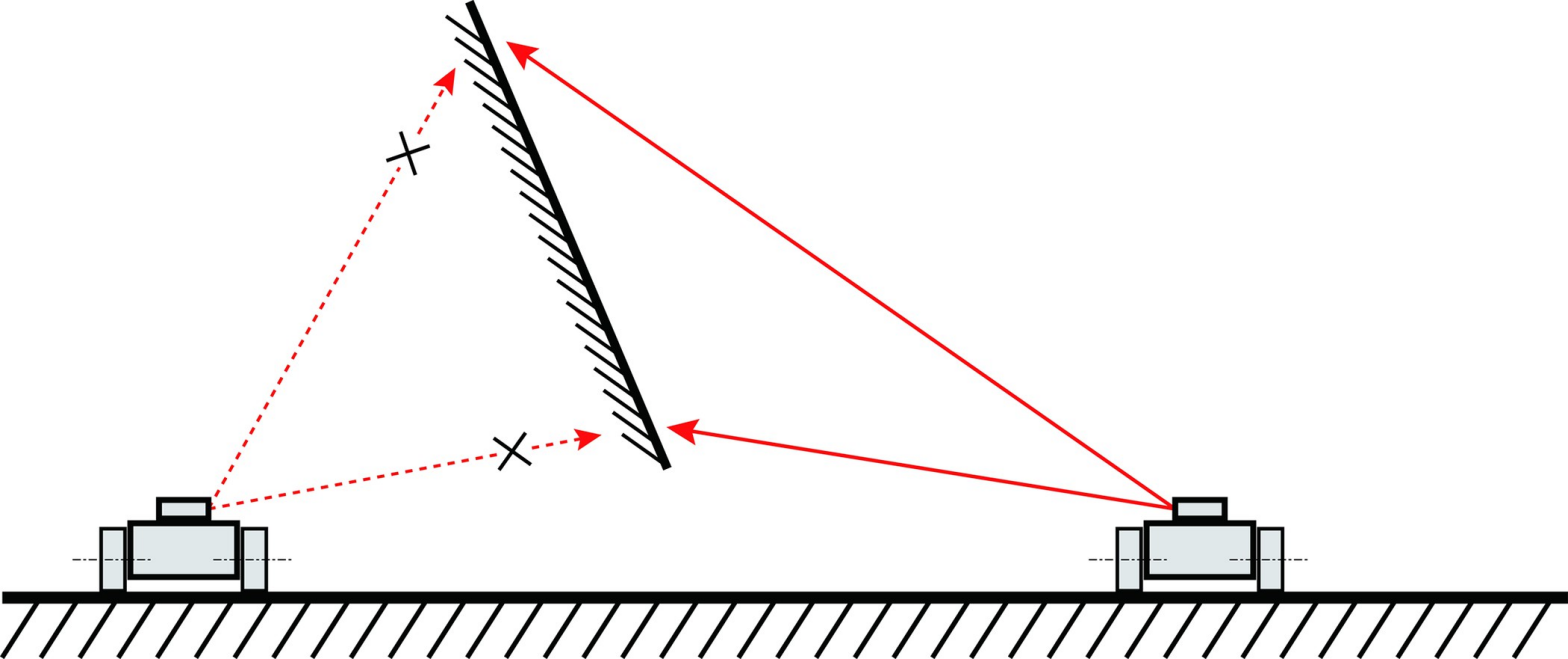
The plane used for positioning is unidirectional.

Based on the ideal plane’s mathematical analysis, the absolute value in Formula (20) renders the Equation subject to both positive and negative possibilities. In other words, the mobile robot can only be situated on one side of the plane or the other. In reality, for any given plane, only one side of the plane surface can be visible, meaning that only one scenario will be considered for the actual observable plane based on stereo vision, as displayed in Fig 7. Consequently, the solution for the absolute value Eq (20) is singular.

Fig 7 demonstrates that the plane’s visibility is unidirectional. Only a mobile robot positioned on the right can observe the target plane surface. Considering constraint Eq (20) as the generalized line function

ax+by+c=0 (21)

where the coefficient *a* is

$$a=\pi_{1}^{G}\sqrt{{\left(\pi_{1}^{R}\right)}^{2}+{\left(\pi_{2}^{R}\right)}^{2}+{\left(\pi_{3}^{R}\right)}^{2}}$$ (22)

the coefficient *b* is

$$b=\pi_{2}^{G}\sqrt{{\left(\pi_{1}^{R}\right)}^{2}+{\left(\pi_{2}^{R}\right)}^{2}+{\left(\pi_{3}^{R}\right)}^{2}}$$ (23)

and the coefficient *c* is

$$c=\pi_{4}^{G}\sqrt{{\left(\pi_{1}^{R}\right)}^{2}+{\left(\pi_{2}^{R}\right)}^{2}+{\left(\pi_{3}^{R}\right)}^{2}}-\delta_{+}\pi_{4}^{R}\sqrt{{\left(\pi_{1}^{G}\right)}^{2}+{\left(\pi_{2}^{G}\right)}^{2}+{\left(\pi_{3}^{G}\right)}^{2}}$$ (24)

The parameter *δ*_+_ represents the plane’s front direction symbol, as discussed in Fig 7. The definition of the direction parameter *δ*_+_ is related to the plane’s function. When $\pi_{4}^{G}>0$ and $\pi_{4}^{R}>0$, and for the plane represented in the ground coordinate system, if the origin point is situated on the observable side, then *δ*_+_ = 1. Otherwise, if the origin point is positioned on the unobservable side, then *δ*_+_ = −1.

The multiple observed planes captured by the camera system comprise the constraint set, as demonstrated in Eq (25).


$$\{\begin{array}{c} a_{1}x+b_{1}y+c_{1}=0\\ \ldots \\ a_{n}x+b_{n}y+c_{n}=0 \end{array}$$ (25)

If there is only one equation, the mobile robot’s position is constrained to a line. If there are two equations, the robot’s position can be calculated using a quadratic system of equations. When there are more than two equations, the mobile robot’s exact position can be determined through a regression function. Assuming that the *x*-coordinate and *y*-coordinate of the mobile robot are independent and the estimations conform to a normal distribution, the least squares method can be employed to obtain an unbiased set of estimates, $\left(\hat{x},\hat{y}\right)$. The position estimation regression statistical model is presented in Eq (26).


$$\varepsilon_{i}(x,y)=d_{i}(x,y)=\frac{a_{i}\hat{x}+b_{i}\hat{y}+c_{i}}{\sqrt{a_{i}^{2}+b_{i}^{2}}}$$ (26)

In Eq (26), *ε*_*i*_(*x*,*y*) represents the statistic sample error, represented by the distance between the estimated coordinates to the constraint line. This equation is valid, as accurate observations should result in all constraint lines intersecting at a single point $\left(\hat{x},\hat{y}\right)$ rendering the distance, *d*_*i*_(*x*,*y*), defined in Eq (26) as zero. The distance error of estimation *ε*_*i*_(*x*,*y*) is considered to follow a normal distribution, $\varepsilon_{i}∼Ν(0,\sigma_{i}^{2})$. The variance of the distribution relies on the quantity of plane observation.

According to the least square method principle, the evaluation function can be expressed as Eq (27).


$$Q(x,y)=\sum_{i=1}^{n}\frac{{\left[\varepsilon_{i}(x,y)\right]}^{2}}{\sigma_{d,i}^{2}}=\sum_{i=1}^{n}{\left(\frac{a_{i}x+b_{i}y+c_{i}}{\sigma_{d,i}\sqrt{a_{i}^{2}+b_{i}^{2}}}\right)}^{2}$$ (27)

The partial derivative of Eq (27) should equal zero, as shown in Eq (28).


$$\left\{ \begin{array}{l} \frac{\partial Q}{\partial x}=2\sum_{i=1}^{n}a_{i}(\frac{a_{i}x+b_{i}y+c_{i}}{\sigma_{d,i}^{2}(a_{i}^{2}+b_{i}^{2})})=0\\ \frac{\partial Q}{\partial y}=2\sum_{i=1}^{n}b_{i}(\frac{a_{i}x+b_{i}y+c_{i}}{\sigma_{d,i}^{2}(a_{i}^{2}+b_{i}^{2})})=0 \end{array}\right.$$ (28)

Upon organizing Eq (28) yields Eq (29).


$$\left\{ \begin{array}{l} \sum_{i=1}^{n}\frac{a_{i}^{2}}{\sigma_{d,i}^{2}(a_{i}^{2}+b_{i}^{2})}x+\sum_{i=1}^{n}\frac{a_{i}b_{i}}{\sigma_{d,i}^{2}(a_{i}^{2}+b_{i}^{2})}y+\sum_{i=1}^{n}\frac{a_{i}c_{i}}{\sigma_{d,i}^{2}(a_{i}^{2}+b_{i}^{2})}=0\\ \sum_{i=1}^{n}\frac{a_{i}b_{i}}{\sigma_{d,i}^{2}(a_{i}^{2}+b_{i}^{2})}x+\sum_{i=1}^{n}\frac{b_{i}^{2}}{\sigma_{d,i}^{2}(a_{i}^{2}+b_{i}^{2})}y+\sum_{i=1}^{n}\frac{b_{i}c_{i}}{\sigma_{d,i}^{2}(a_{i}^{2}+b_{i}^{2})}=0 \end{array}\right.$$ (29)

The solution of Eq (29) is shown in Eq (30)

$$\left\{ \begin{array}{l} x=\frac{\sum_{i=1}^{n}\frac{a_{i}b_{i}}{\sigma_{d,i}^{2}(a_{i}^{2}+b_{i}^{2})}\sum_{i=1}^{n}\frac{b_{i}c_{i}}{\sigma_{d,i}^{2}(a_{i}^{2}+b_{i}^{2})}-\sum_{i=1}^{n}\frac{a_{i}c_{i}}{\sigma_{d,i}^{2}(a_{i}^{2}+b_{i}^{2})}\sum_{i=1}^{n}\frac{b_{i}^{2}}{\sigma_{d,i}^{2}(a_{i}^{2}+b_{i}^{2})}}{\sum_{i=1}^{n}\frac{a_{i}^{2}}{\sigma_{d,i}^{2}(a_{i}^{2}+b_{i}^{2})}\sum_{i=1}^{n}\frac{b_{i}^{2}}{\sigma_{d,i}^{2}(a_{i}^{2}+b_{i}^{2})}-\sum_{i=1}^{n}\frac{a_{i}b_{i}}{\sigma_{d,i}^{2}(a_{i}^{2}+b_{i}^{2})}\sum_{i=1}^{n}\frac{a_{i}b_{i}}{\sigma_{d,i}^{2}(a_{i}^{2}+b_{i}^{2})}}\\ y=\frac{\sum_{i=1}^{n}\frac{a_{i}c_{i}}{\sigma_{d,i}^{2}(a_{i}^{2}+b_{i}^{2})}\sum_{i=1}^{n}\frac{a_{i}b_{i}}{\sigma_{d,i}^{2}(a_{i}^{2}+b_{i}^{2})}-\sum_{i=1}^{n}\frac{a_{i}^{2}}{\sigma_{d,i}^{2}(a_{i}^{2}+b_{i}^{2})}\sum_{i=1}^{n}\frac{b_{i}c_{i}}{\sigma_{d,i}^{2}(a_{i}^{2}+b_{i}^{2})}}{\sum_{i=1}^{n}\frac{a_{i}^{2}}{\sigma_{d,i}^{2}(a_{i}^{2}+b_{i}^{2})}\sum_{i=1}^{n}\frac{b_{i}^{2}}{\sigma_{d,i}^{2}(a_{i}^{2}+b_{i}^{2})}-\sum_{i=1}^{n}\frac{a_{i}b_{i}}{\sigma_{d,i}^{2}(a_{i}^{2}+b_{i}^{2})}\sum_{i=1}^{n}\frac{a_{i}b_{i}}{\sigma_{d,i}^{2}(a_{i}^{2}+b_{i}^{2})}} \end{array}\right.$$ (30)

where $\sigma_{d,i}^{2}$ represents the distance observation variance for plane *i*, which is influenced by various factors. In this case, the variance is considered to have an inversely proportional relationship depicted in Eq (31).

$$\sigma_{d,i}^{2}=\frac{\sigma_{P,d}^{2}}{N}$$ (31)

where *N* represents the point cloud size characterizing the observed plane. And $\sigma_{P,d}^{2}$ represents the variance of a single point distance measurement. A larger point cloud size results in a smaller variance.

The pose angle estimation process resembles the mobile robot position estimation. The pose angle is defined as the included angle, *φ*, between the normal vector of plane *π*^G^ projected onto the plane of *xOy*, and the normal vector *π*^R^ projected onto the plane of *xOy*. The included angle adheres to the Eq (32).

$$\left[\begin{array}{c} \pi_{1}^{G}\\ \pi_{2}^{G} \end{array}]=[\begin{array}{cc} \cos\varphi & \sin\varphi \\ -\sin\varphi & \cos\varphi \end{array}][\begin{array}{c} \pi_{1}^{R}\\ \pi_{2}^{R} \end{array}\right]$$ (32)

and the included angle can be expressed as (33).


$$\varphi =\arccos\frac{\pi_{1}^{G}\pi_{1}^{R}+\pi_{2}^{G}\pi_{2}^{R}}{{\left(\pi_{1}^{R}\right)}^{2}+{\left(\pi_{2}^{R}\right)}^{2}}$$ (33)

According to the observation Eq (33), a single plane constraint suffices for pose angle estimation, whereas position estimation necessitates two plane constraints.

The precondition of Eq (33) is that the plane *π*^G^ is not parallel to the ground, which corresponds to the *xOy* plane of ground coordinate system. This condition is relatively simple to satisfy in actual mobile robot localization scenario. Firstly, plane observation errors ensure that the statistical probability of measuring a ground-parallel plane is zero. Secondly, mobile robot cameras are typically mounted on the robot’s side, making it nearly impossible to observe a ground-parallel plane unless the line of sight is directed upwards. Lastly, prior information can be used to eliminate ground-parallel planes.

The weighted error sum of the square equation is presented in Eq (34).


$$Q(\varphi )=\sum_{i=1}^{n}\frac{{\left(\varphi -\varphi_{i}\right)}^{2}}{\sigma_{\varphi ,i}^{2}}$$ (34)

The least squares estimation follows that

$$Q(\hat{\varphi })=\underset{\varphi }{\min}Q(\varphi )$$ (35)

The weighted coefficient for the pose angle estimation is

$$\sigma_{\varphi ,i}^{2}=\frac{\sigma_{P,\varphi }^{2}}{N}$$ (36)

The normal Equation for the least square estimation is

$$\frac{\partial Q}{\partial \varphi }=2\sum_{i=1}^{n}\frac{\varphi -\varphi_{i}}{\sigma_{\varphi ,i}^{2}}=0$$ (37)

Upon organizing Eq (37), the weighted least square estimation for the pose angle is depicted in Eq (38)

$$\varphi =\frac{\sum_{i=1}^{n}\frac{\varphi_{i}}{\sigma_{\varphi ,i}^{2}}}{\sum_{i=1}^{n}\frac{1}{\sigma_{\varphi ,i}^{2}}}$$ (38)

## 4 Experiment validation of the proposed method

### 4.1 Prototype development and workspace configuration

**Fig 8:**
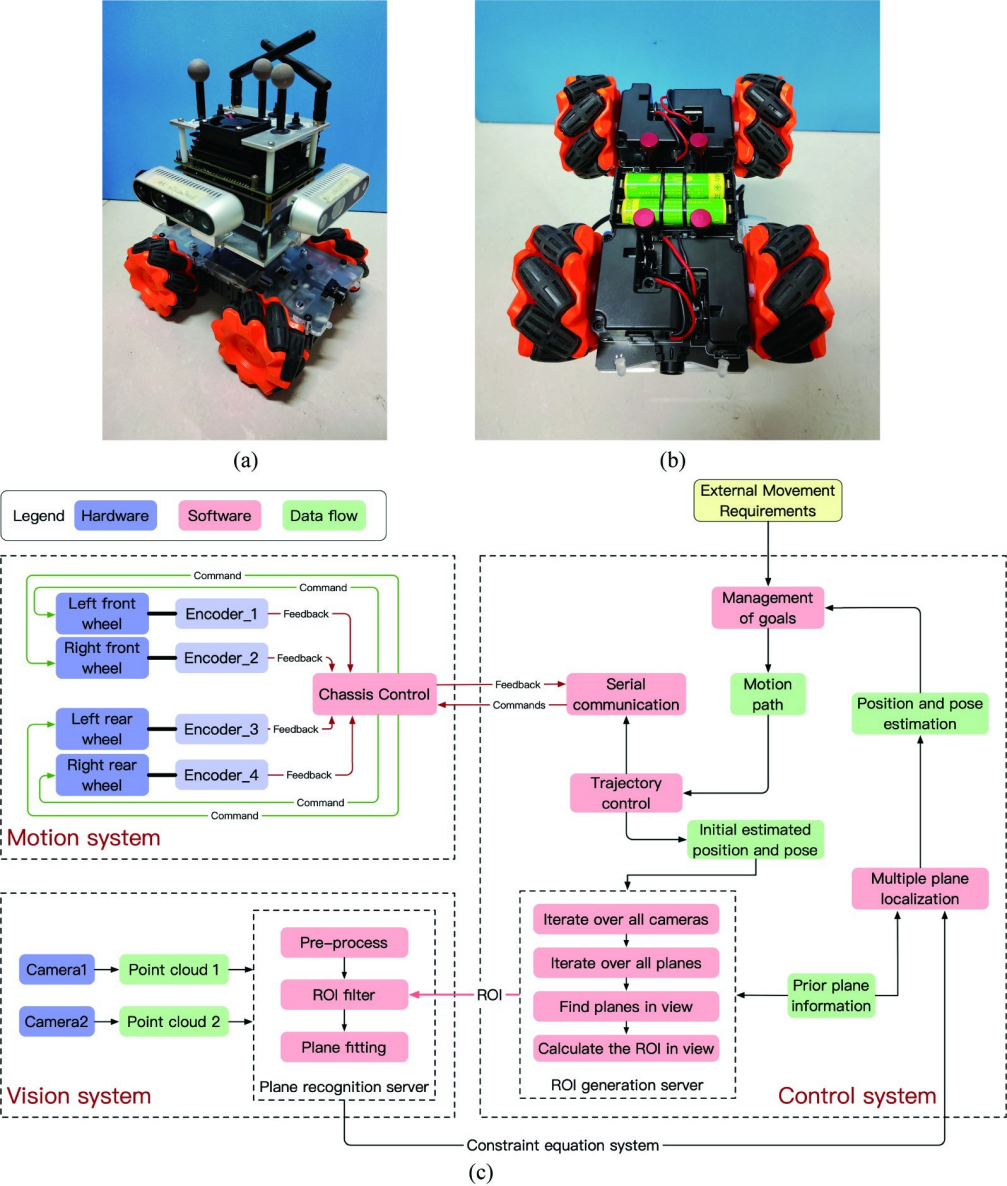
Prototype of the mobile robot system. (a)Overall view of the mobile robot prototype. (b)The four Mecanum wheels of the mobile robot. (c)The structure of the mobile robot system.

The prototype’s appearance is displayed in Fig 8(A). Mobile robot consists of vision, motion, and control systems. The vision system includes two Intel RealSense D435i RGB-D cameras, while the motion system is an omnidirectional mobile chassis primarily composed of four Mecanum wheels, as shown in Fig 8(B). The central control system mainly consists of NVIDIA Jetson Nano modules. The structure of the mobile robot system is illustrated in Fig 8(C), where blue blocks represent hardware components, the red blocks represent software components, and the green blocks represent data flow components. MATLAB is used for the analysis of the experimental result’s initial data and figure drawing.

The symmetrically arranged Mecanum wheels enable multidirectional movement of the chassis, with encoder feedback enhancing movement precision. The motion system communicates with the control system via a standard serial port communication protocol.

The control system receives external movement requirements specifying the macroscopic motion of the mobile robot. The motion target management module processes these requirements into motion paths and forwards them to the motion trajectory control module. This module calculates specific linear and rotational motion parameters for the current motion stage and transmits them to the serial communication module in a formatted structure. The serial communication module sends motion commands to the motion system in accordance with the serial communication protocol and monitors the chassis’s movement.

The control system’s pivotal module is the ROI generation server. It iterates through all cameras and structured planes based on initial estimated coordinates, calculating filter ROIs from the theoretically visible plane set. The vision system filters point clouds for each plane, constraining the mobile robot’s position and pose. Each filtered ROI is sent to the plane recognition server, which establishes the constraint equation system.

The vision system identifies parameters of each constraint plane within its field of view and transmits the constraint equation system to the multi-plane positioning module. In conjunction with the known structured plane set, position and pose constraints for the mobile robot are obtained.

Eqs (39) and (40) display the homogeneous transformation matrix from the two camera coordinate systems to the robot coordinate system. Eq (39) exemplifies the homogeneous transformation matrix for the camera located directly in front of the mobile robot.


$$T_{C,1}^{R}=[\begin{array}{cccc} 0 & 0 & 1 & 82.85\\ 1 & 0 & 0 & 17.5\\ 0 & 1 & 0 & 135\\ 0 & 0 & 0 & 1 \end{array}]$$ (39)

The first camera’s non-unit rotation matrix in the top-left transformation matrix results from the *z*-axis in the camera coordinate system pointing directly forward, and the camera coordinate system’s origin being situated on the camera’s left infrared sensing element, causing a 17.5mm *y*-direction offset.

Eq (40) presents the homogenous transformation for the second camera.


$$T_{C,2}^{R}=[\begin{array}{cccc} 1 & 0 & 0 & 17.5\\ 0 & 0 & 1 & -75.85\\ 0 & 1 & 0 & 135\\ 0 & 0 & 0 & 1 \end{array}]$$ (40)

**Fig 9:**
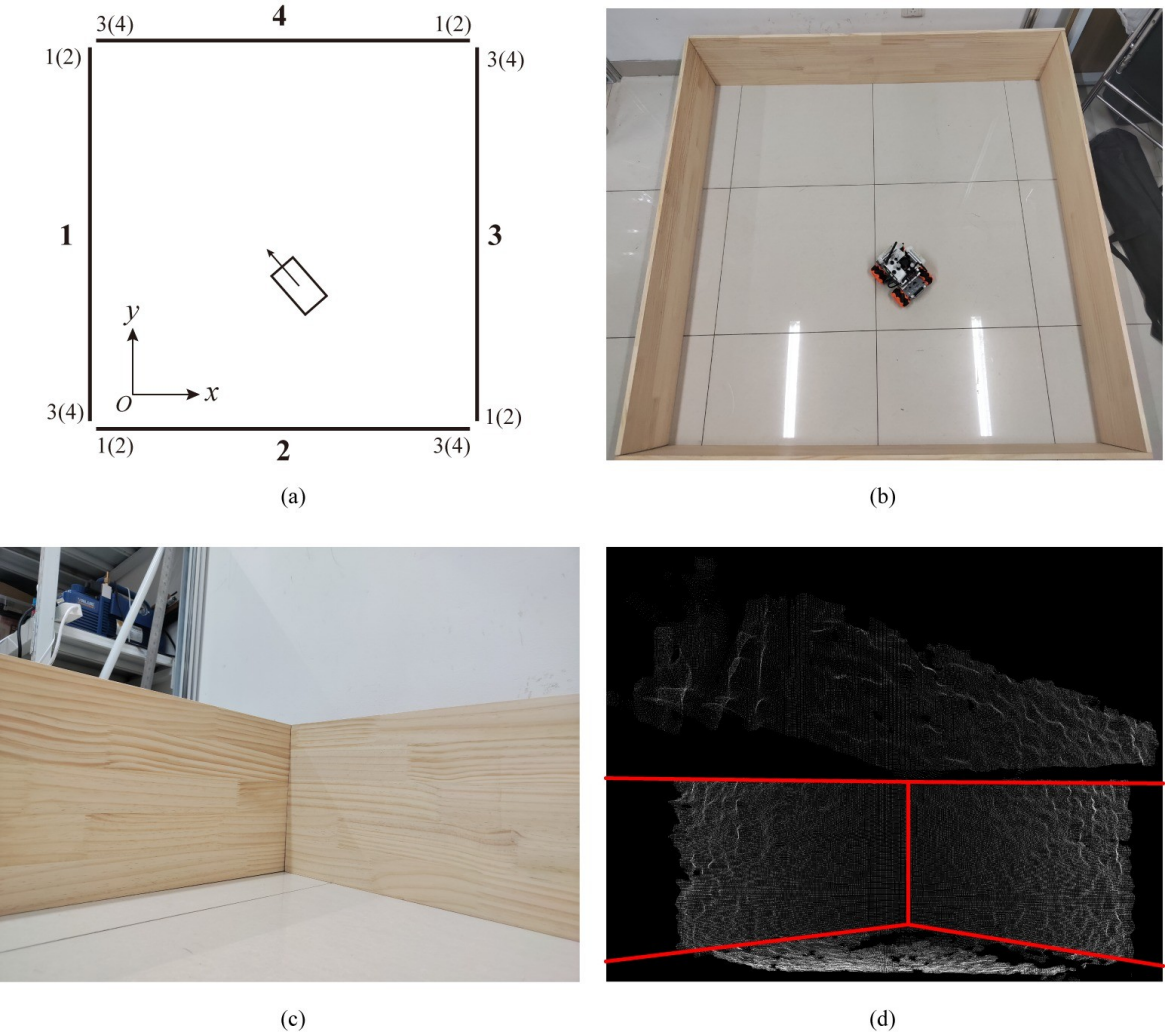
The layout of the experiment workspace and the point cloud example for localization. (a)Experimental workspace and the identified numbers of the planes and their vertices. (b)Overall view of the workspace. (c)Example of observed planes in view. (d)Example of the filtered ROIs when identifying the planes.

The mobile robot test site is encircled by 300mm-high walls, forming a 1500 mm by 1500 mm area, as demonstrated in Fig 9. The experimental workspace simulates a building’s wall, which the mobile robot employs for indoor localization.

Fig 9(a) displays the wall numbers utilized for visual positioning and corresponding vertex numbers representing the wall plane. Numbered in a clockwise manner, the ground coordinate system’s origin is positioned within the workspace, with the bottom-left point in Fig 9(A) having coordinates (-250, -250). The real-world experimental setup is shown in Fig 9(B), while Fig 9(C) exemplifies one camera’s visible area for localization. Planes used for localization are segmented by ROI filters, as seen in Fig 9(D).

Table 2 contains the structural parameters of each plane within the ground coordinate system.

**Table 2:** The structured parameters of a-priori planes.

Plane seq.	Vertex seq.	Coordinates for the vertices		
		*x*	*y*	*z*
**1**	1	-250	1250	300
	2	-250	1250	0
	3	-250	-250	300
	4	-250	-250	0
**2**	1	-250	-250	300
	2	-250	-250	0
	3	1250	-250	300
	4	1250	-250	0
**3**	1	1250	-250	300
	2	1250	-250	0
	3	1250	1250	300
	4	1250	1250	0
**4**	1	1250	1250	300
	2	1250	1250	0
	3	-250	1250	300
	4	-250	1250	0

The ground coordinate system’s *xOy* plane coincides with the surface on which the mobile robot operates. Similarly, the origin of the robot coordinate system is situated on the same plane where the mobile robot moves.

### 4.2 Robustness testing of proposed method with various initial position and pose

Exist difference between the mobile robot’s estimated initial coordinates and the actual coordinates, potentially lead to some points in the point cloud not representing the relevant plane. Nevertheless, the RANSAC method exhibits high tolerance for outliers in the point cloud, ensuring minimal impact on plane parameter identification results even with numerous outlier points within the filter ROI. This experimental design aims to verify this robustness.

The mobile robot’s center is positioned at coordinates (500, 500), with its direction vector represented in the ground coordinate system as (-1, 0) and its pose angle as π. The pose angle is defined as the angle between the mobile robot’s direction vector and the ground coordinate system’s *x*-axis.

Initially, the mobile robot is placed facing the target plane, with the target plane’s normal vector parallel to the camera’s *z*-axis. The *x*-coordinate, *y*-coordinate, and pose angle values are adjusted around the actual value.

Subsequently, an initial pose angle error of π/6 is manually added on the actual pose angle. The actual pose angle of the mobile robot remains 5π/6, and the assumed pose and position variations are consistent with the first set of experiments.

While keeping the robot stationary, the initial estimated position and pose are adjusted. Two primary indicators are evaluated to assess the impact on plane identification: the size of the point cloud filtered by the calculated ROI and the successful identification of the corresponding plane within the point cloud. To ensure point cloud size accuracy, no down-sampling is performed during pre-processing.

**Fig 10:**
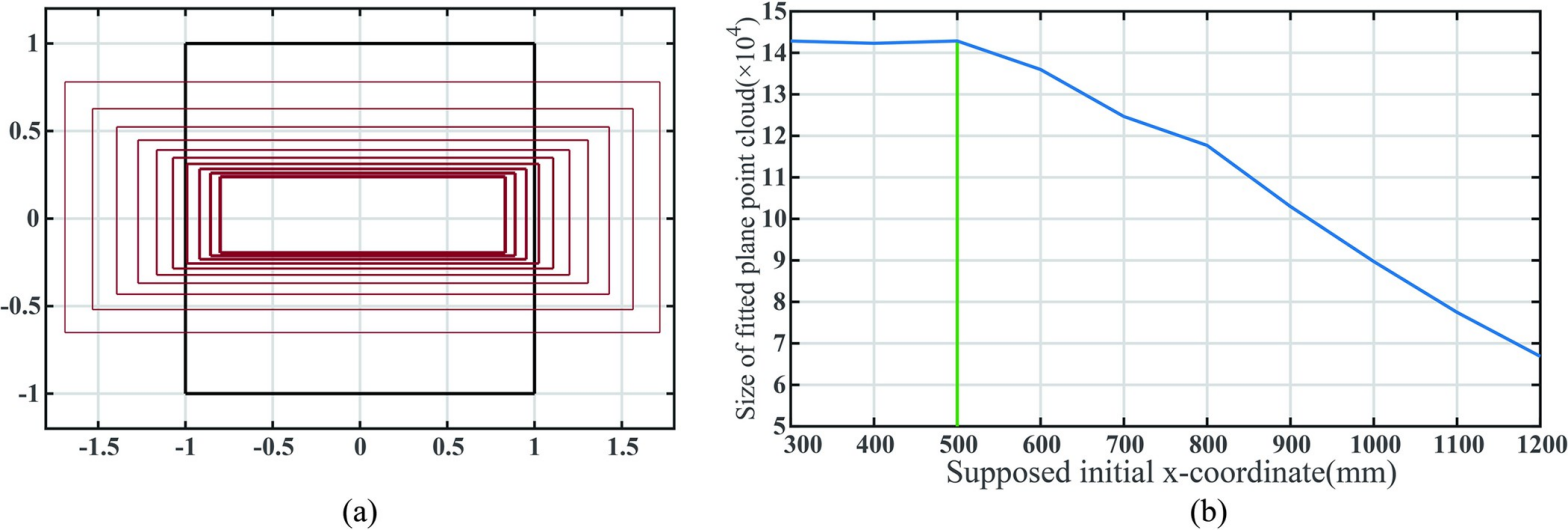
(a)The change of the plane region to the view field region (VFR). (b)Size of point cloud inside filter ROI when the supposed initial *x*-coordinate of the mobile robot changes.

The assumed initial *x*-coordinate of the mobile robot ranges from 200mm to 1200mm. Fig 10(A) illustrates the variation of the filtered ROI within the extended image coordinate system, while Fig 10(B) displays the curve of the number of fitted plane points in the point cloud obtained by filter ROI as the initial *x*-coordinate.

As shown in Fig 10(A), the black rectangle represents the boundary of the view field region (VFR), while the red rectangles indicate the filtered ROIs of the corresponding planes. Larger line widths signify greater assumed initial distances between the camera and the target plane.

The green vertical line in Fig 10(B) corresponds to the actual x-coordinate of the robot. As the assumed initial distance between the mobile robot and the plane increases, the filter ROI area and the number of plane fitting points obtained after point cloud screening decrease. However, the identification remains successful even as the assumed distance between the camera and the target plane increases from 300mm to 1500mm. A larger filtered ROI does not always correspond to a more extensive plane point cloud, as the ROIs already contain all the point clouds representing the plane within the field of view.

**Fig 11:**
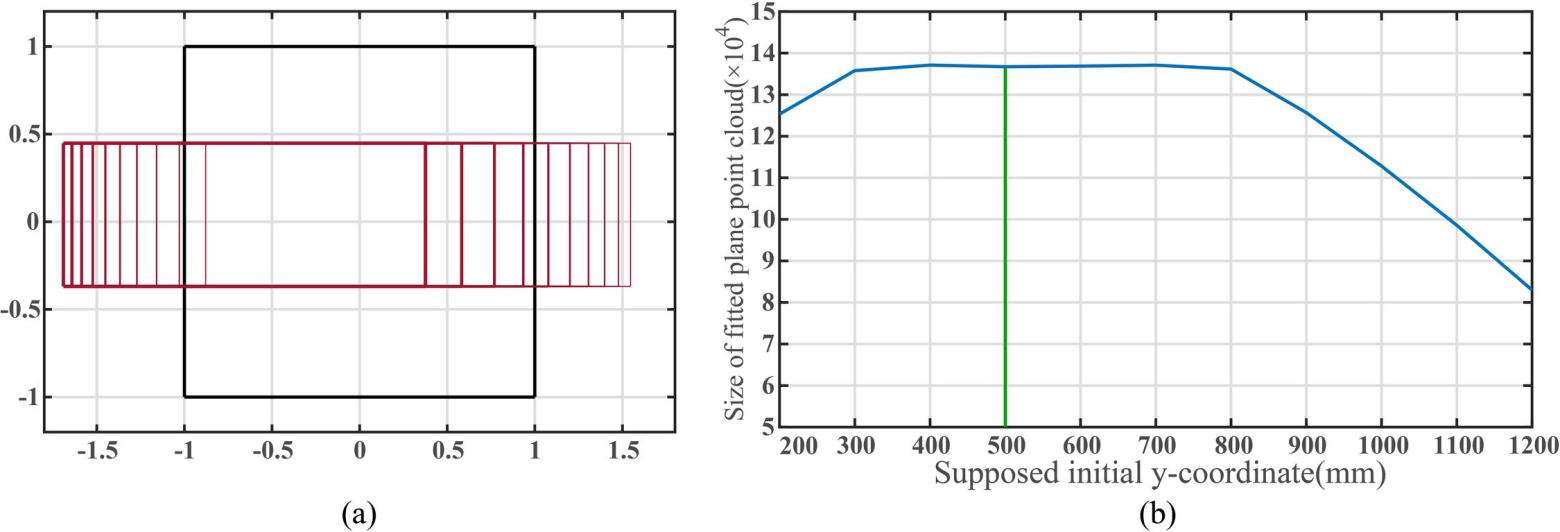
(a)The change of the plane region to the view field region (VFR). (b)Size of point cloud inside filter ROI when the supposed initial *y*-coordinate of the mobile robot changes.

Fig 11(A) demonstrates the geometric changes in the filtered ROI as the assumed initial y-coordinate varies within the extended image coordinate system, and Fig 11(B) presents the curve of fitted plane points as the initial y-coordinate increases from 200mm to 1200mm.

As shown in Fig 11(A), varying the assumed initial y-coordinate influences the horizontal location of the rectangle ROI. When the assumed initial y-coordinate is equal to the actual y-coordinate, the number of fitted plane points reaches its maximum. As the assumed initial y-coordinate increases, the actual number of identified plane points first increases and then decreases.

**Fig 12:**
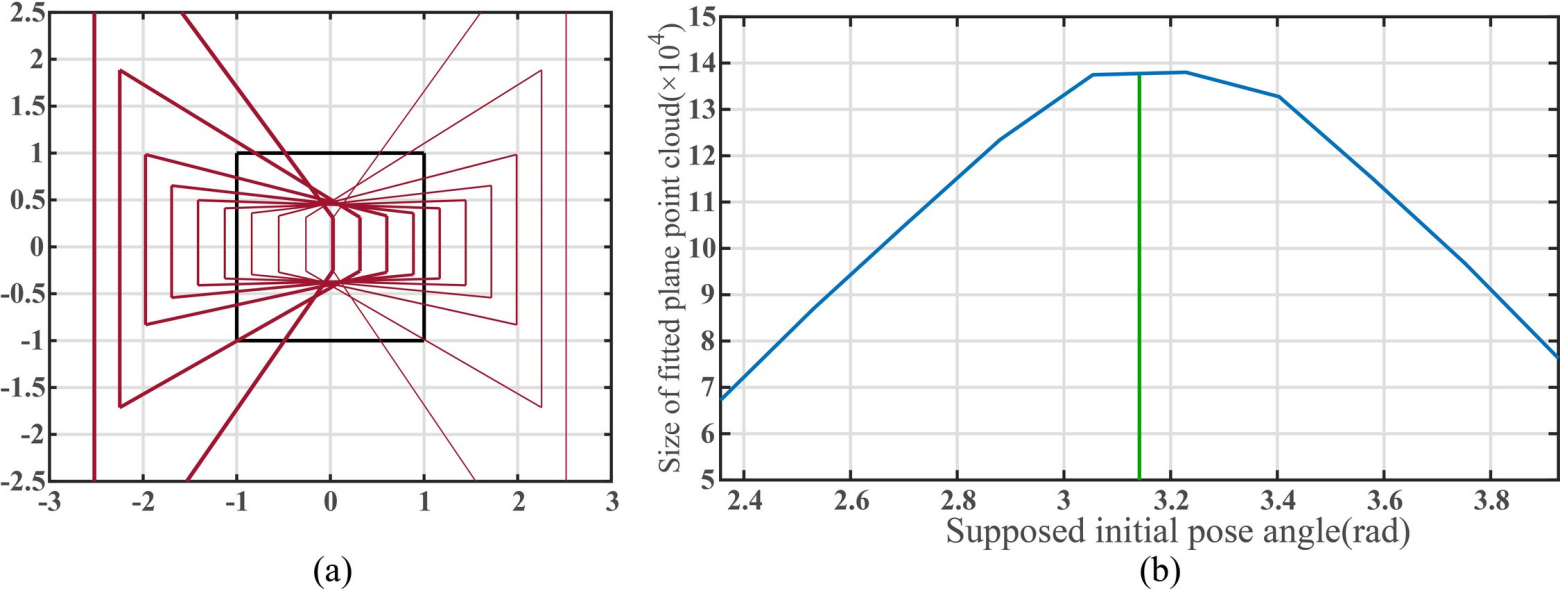
(a)The change of the plane region to the view field region (VFR). (b)Size of point cloud inside filter ROI when the supposed initial pose angle of the mobile robot changes.

Fig 12(A) reveals the geometric changes in the filtered ROI as the assumed initial pose varies within the extended image coordinate system, and Fig 12(B) shows the fitted plane points number curve as the initial pose angle increases from 2π/3 to 4π/3. The assumed initial coordinates of mobile robot are (500, 500).

The plane fitting result in Fig 12(B) indicate that the number of fitted plane points initially increases and then decreases as the pose angle increases. When the camera faces directly toward the target plane, the point cloud reaches its maximum.

When the camera’s *z*-axis is parallel to the plane’s normal vector, the initial position and pose accuracy requirements are not stringent. As seen that in Figs 10–12, even when the assumed initial position and pose undergo significant changes, the target planes can be correctly identified.

In the following experiments, the actual pose angle of the mobile robot is changed, and the camera’s forward direction is not parallel to the normal vector of the plane. The center of the mobile robot is placed at coordinates (500, 500), and its direction vector in the ground coordinate system is $\left(-\sqrt{2}/2,\sqrt{2}/2\right)$. The actual pose angle for the mobile robot is approximately 5π/6. The supposed initial pose is still set as π.

**Fig 13:**
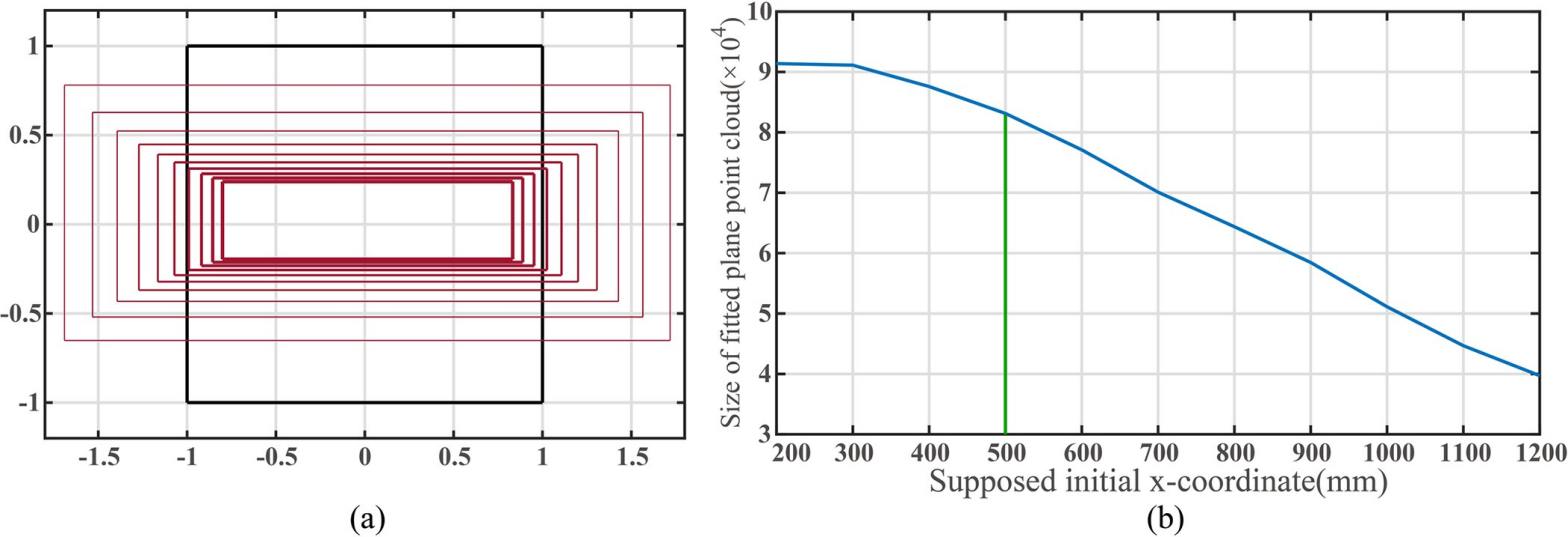
(a)The change of the plane region to the view field region (VFR). (b)Size of point cloud inside filter ROI when the supposed initial *x*-coordinate of the mobile robot changes.

Fig 13(A) demonstrates the geometric changes in the filtered ROI as the supposed initial *x*-coordinate varies within the extended image coordinate system. Fig 13(B) presents the curve of fitted plane points as the initial *x*-coordinate increases from 200mm to 1200mm. The number of fitted plane points decreases as the assumed initial *x*-coordinate increases, but in all experiments, the target planes are correctly identified.

**Fig 14:**
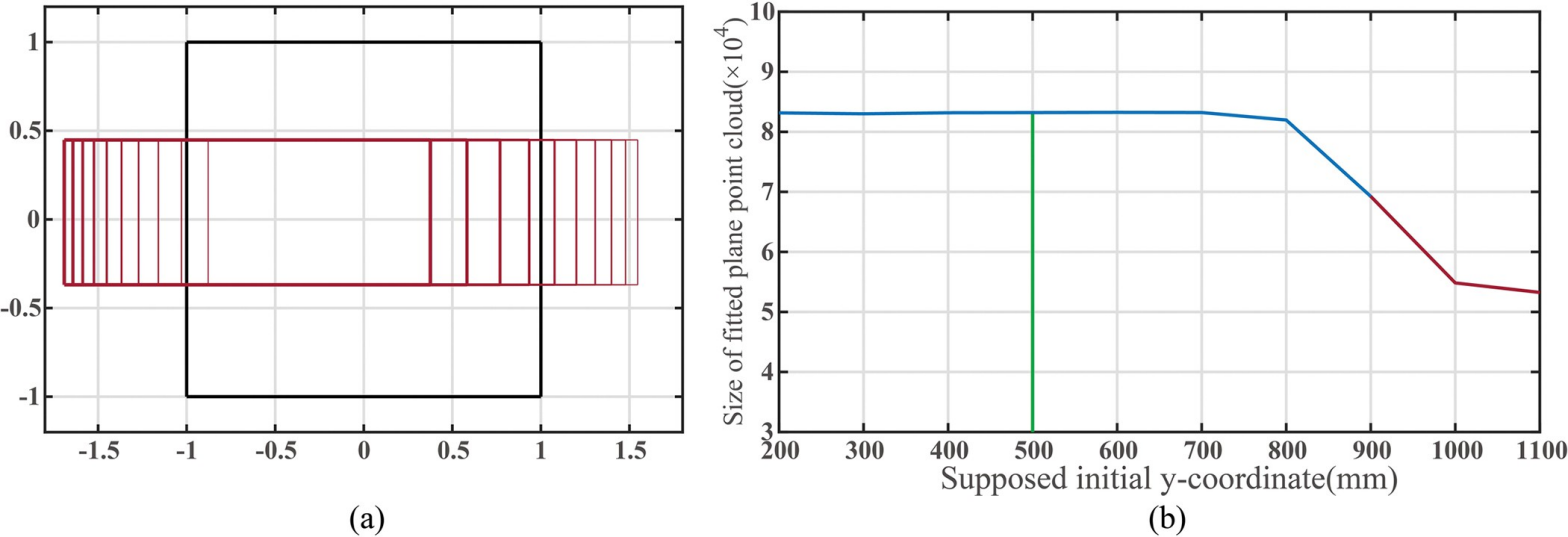
(a)The change of the plane region to the view field region (VFR). (b)Size of point cloud inside filter ROI when the supposed initial *y*-coordinate of the mobile robot changes.

Fig 14(A) displays the geometric changes in the filtered ROI as the assumed initial y-coordinate varies within the extended image coordinate system, and Fig 14(B) shows the curve of fitted plane points as the initial y-coordinate increases from 200mm to 1200mm.

The red segments in Fig 14(B) represent the incorrect identification results of the target plane. As the supposed initial y-coordinate increases, the filter ROI rectangle moves left in the extended image coordinate system. However, the plane on the left in the actual view field is not the target plane.

**Fig 15:**
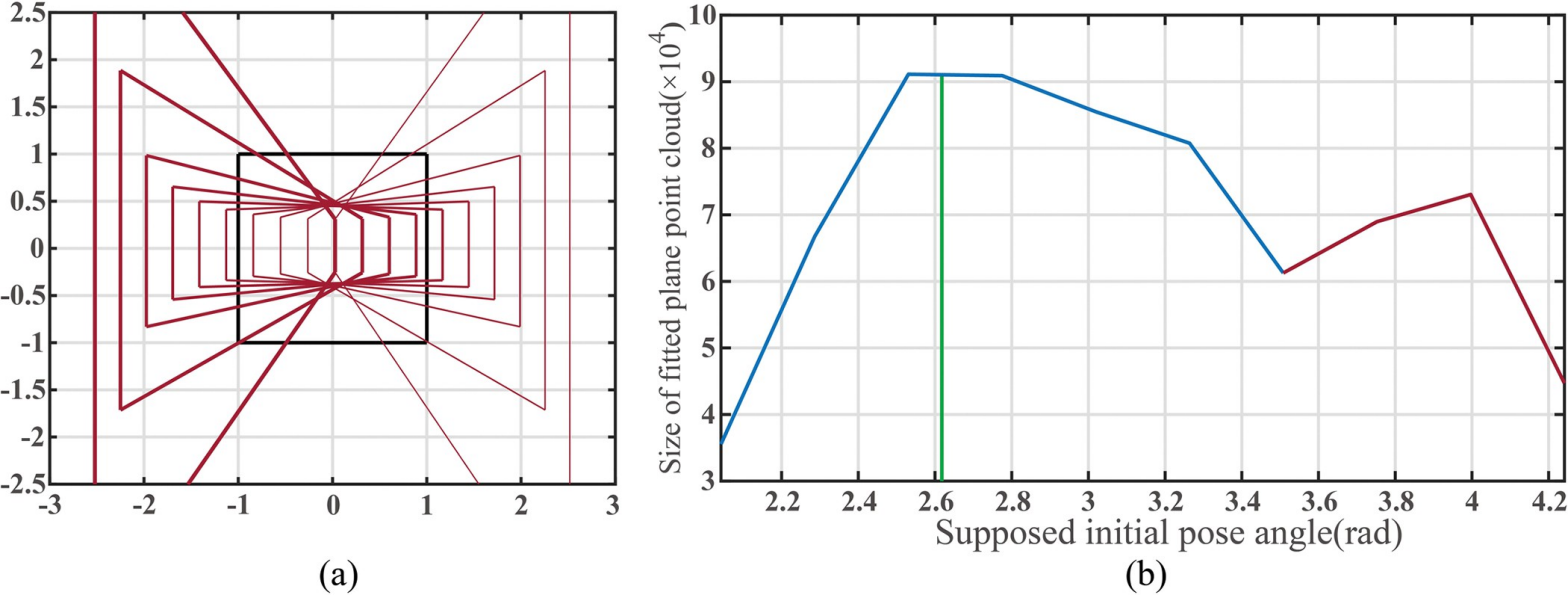
(a)The change of the plane region to the view field region (VFR). (b)Size of point cloud inside filter ROI when the supposed initial pose angle of the mobile robot changes.

Fig 15(A) illustrates the geometric changes in the filtered ROI as the assumed initial y-coordinate varies within the extended image coordinate system, while Fig 15(B) shows the fitted plane points number curve with increasing the supposed initial pose angle from 2π/3 to 4π/3.

The wrong plane represented by red lines is fitted because it occupies a larger area in the camera’s view field. However, as long as the error in the initial pose angle is not too significant, the proposed method can identify the correct plane.

These experiments validate the proposed method based on RANSAC plane fitting and prior plane information. The point cloud of the plane used for localization filtered by ROI allows for a certain degree of initial position and pose error. As long as the estimated initial position and pose of the mobile robot are not too different from the actual pose, the filtered ROI can successfully identify the plane.

### 4.3 Localization validation experiment

**Fig 16:**
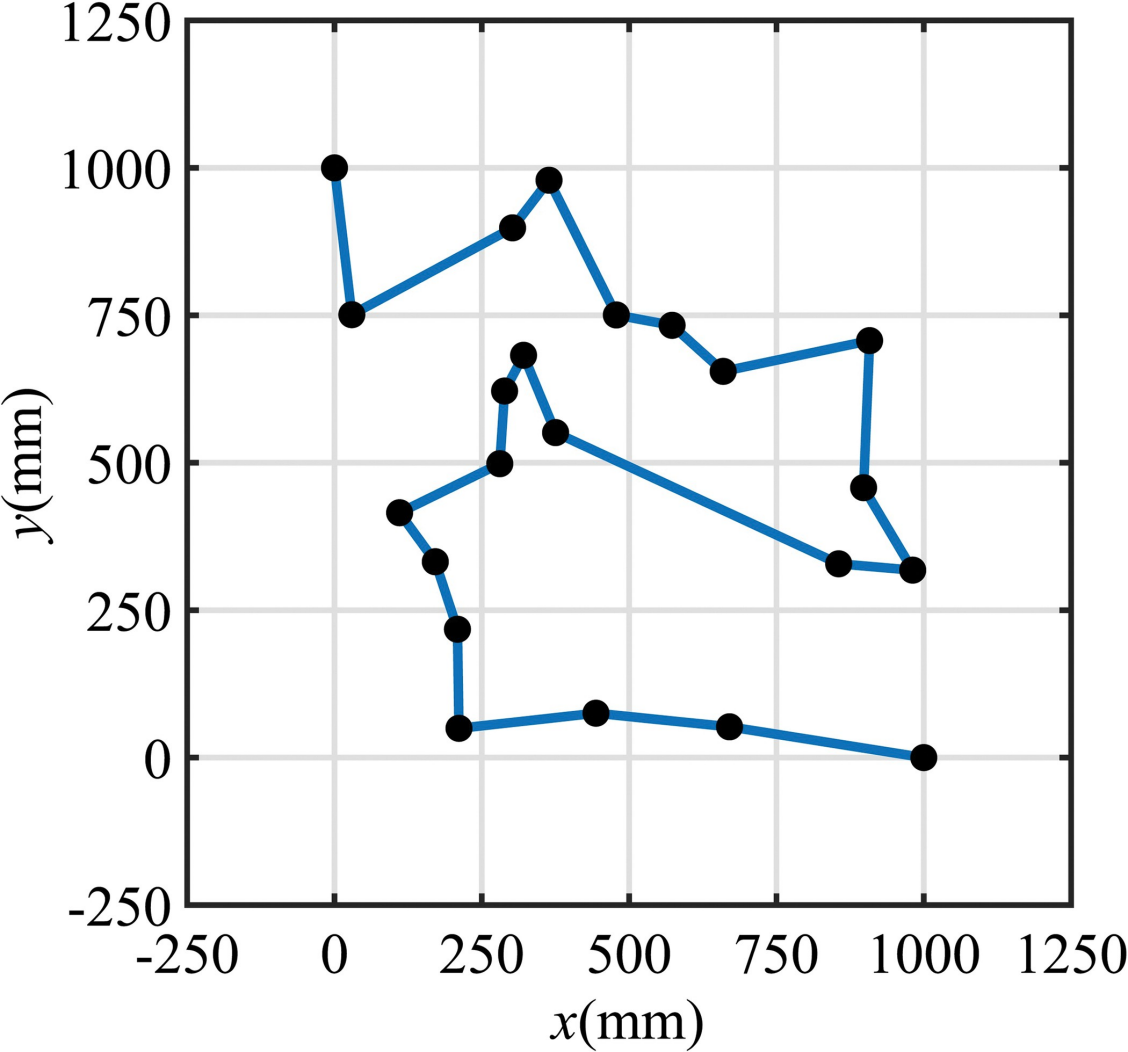
Path sequence of tracking experiment.

An experiment is conducted to ascertain the efficacy of the localization method using scene prior information for visual path tracking in a mobile robot navigation application. The experiment utilizes the former introduced existing prototype and testing environment, where target points are randomly arranged throughout the workspace. The mobile robot starts at the coordinate (0, 1000) and sequentially passes through every target point before ultimately arriving at the coordinate (1000, 0). The sequence of target points is illustrated in Fig 16.

Throughout the pathway execution, the mobile robot employs the aforementioned localization method, which integrates the a-priori information of the environment. At each moment, the robot uses its theoretical pose and position coordinates as initial inputs to calculate the ROI parameters. The obtained ROI parameters are then utilized to filter the plane point clouds within the field of view. The robot subsequently matches the filtered point clouds with the corresponding planes in the a-priori map, which in turn allows for the localization of the mobile robot.

**Fig 17:**
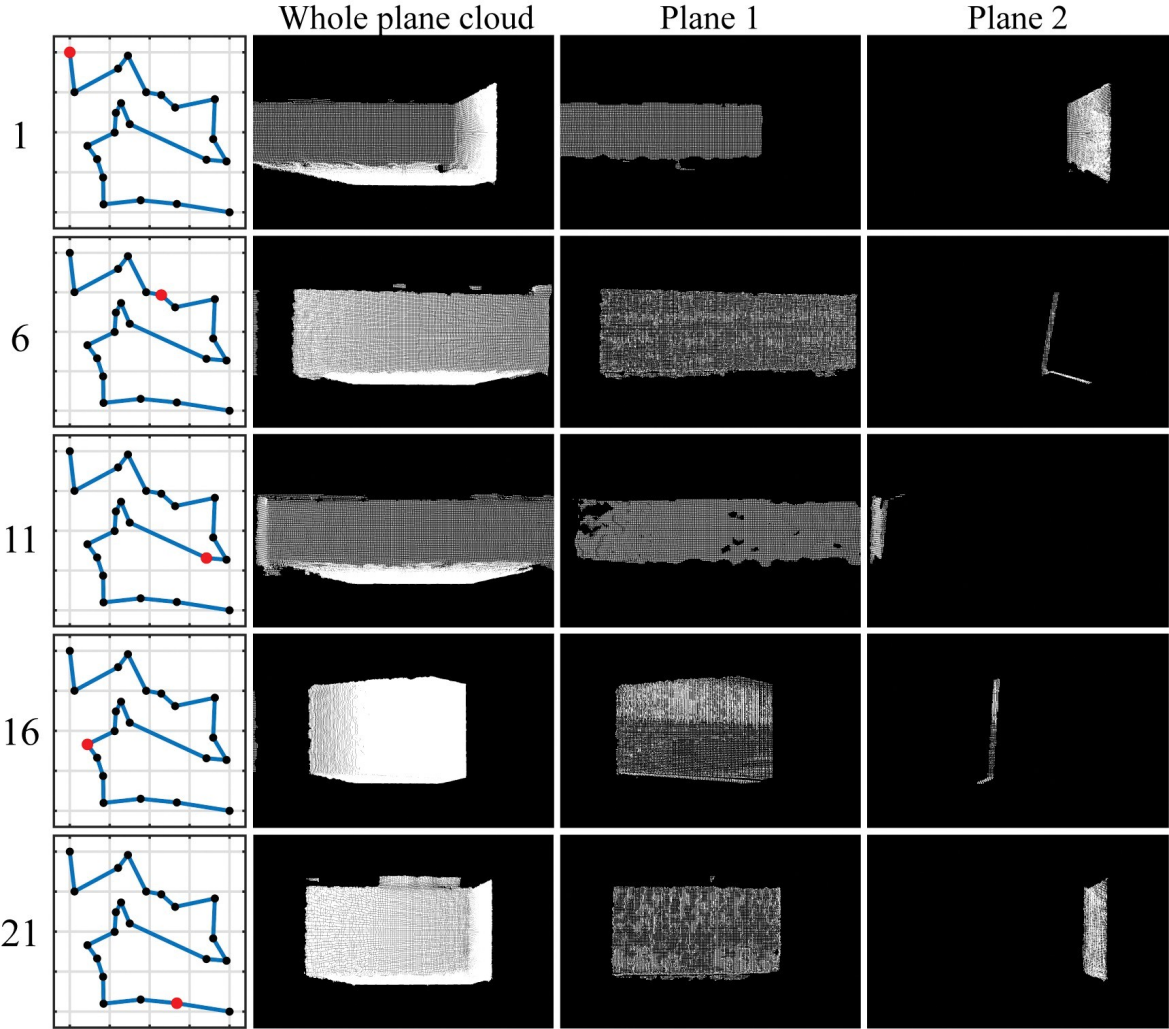
Point cloud segmentation in the process of path tracing.

Fig 17 exhibits the scene point clouds captured by the forward-facing camera during the path tracking process. The figure also illustrates the plane point clouds obtained through segmentation and plane fitting, which served localization purposes.

As seen in Fig 17, there are instances where the forward-facing camera can only observe one plane. However, with the help of side-mounted cameras, the system can consistently observe two or more planes in the process of path tracing. By comparing the localization errors before and after implementing the visual tracking navigation method with scene prior information, it is found that using the a-priori plane information for localization significantly reduces the motion error. This improvement suggests that the proposed method can effectively perform the localization tasks associated with featureless or repetitive environments, leading to better overall performance.

**Fig 18:**
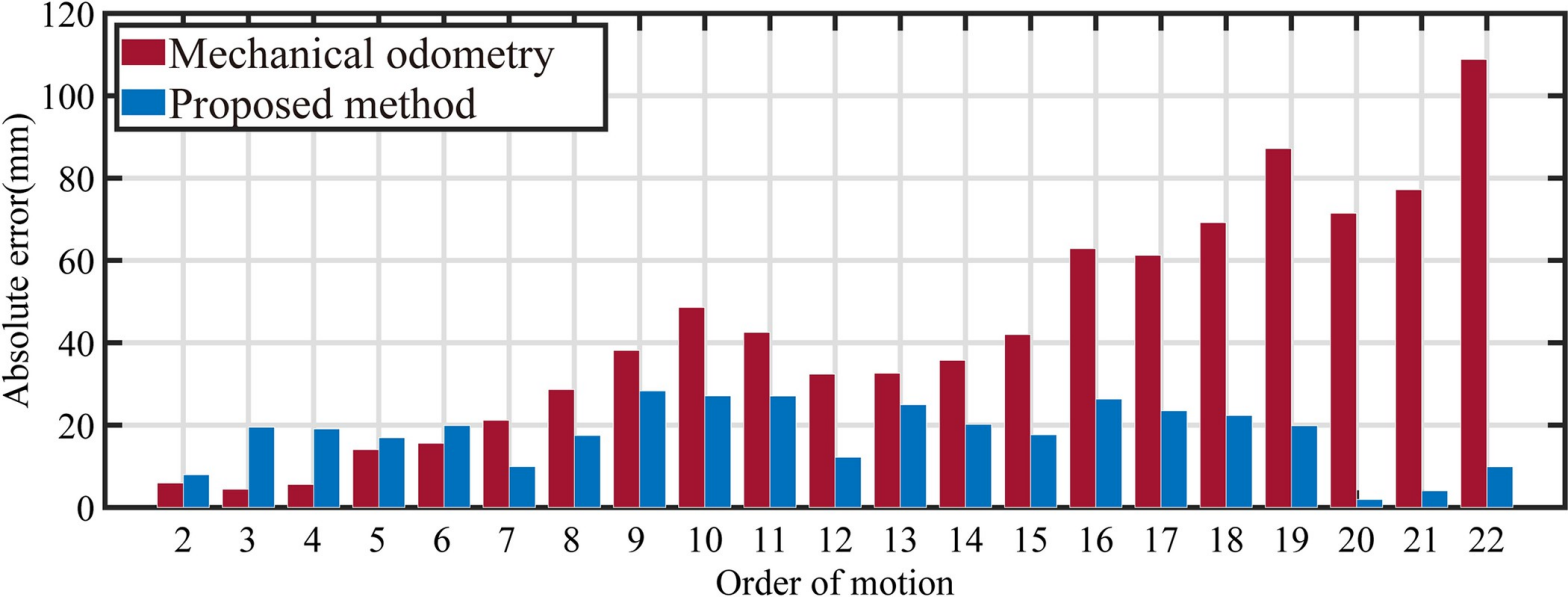
Comparison before and after using proposed method.

Specific experimental results, using the point cloud segmentation method based on a-priori plane, are illustrated in Fig 18. For the comparison experiment, the path presented in Fig 16 is executed with and without visual feedback, and each experiment is repeated 15 times. The average coordinates for each path and position are calculated and compared to the ideal coordinates to determine the offset distance between the actual and ideal positions. The actual position of the mobile robot is accurately captured using a motion capture system with a precision better than 1mm, which can be considered as the ground truth compared to the vision system. Since the first point is manually positioned, the error for this point is insignificant, and the comparison starts from the second point. The numerical data is available by S1 Table.

In the figure above, the localization errors at various points along the path for both cases, with and without visual feedback, are presented. It is evident that the motion error accumulates when visual feedback is not utilized. The motion error is reduced when visual feedback is employed. The comparative analysis of the experiment results is shown in Table 3.

**Table 3:** Results of the comparative experiment.

	MAE (mm)	SD (mm)	RMSE (mm)
**Mechanical odometry**	41.15	29.52	50.25
**Proposed method**	17.77	7.50	19.22

Based on the table results, it can be preliminary concluded that the proposed localization method is effective.

## 5 Discussion

A-priori planes may be acquired, for instance, from a building’s blueprint in BIM or from a pre-existing map in SLAM. These planes are represented by their vertices. As length or height increases, some vertices and edges of the a-priori plane may vanish from the current perspective, rendering only a partial area visible. Nevertheless, vertices beyond the field of view can be arranged in the suggested extended image coordinate system.

Similar to several other approaches, the localization procedure necessitates an initial pose as a starting parameter for the algorithm [36, 40, 41]. Contrasting with the plane extraction technique in [27, 28], the method proposed herein capitalizes on known map information to extract plane point clouds. The method introduced in [30] should meet the criteria that planes be parallel or perpendicular to one another, a strict criterion that restricts localization application. Within numerous well-designed structures, corridors are not always aligned horizontally and vertically. The proposed method in this article provides the analytical solution for localization with planes of any pose. Moreover, the number of planes is not limited. The pose and position estimations are based on the weighted least squares. If one observed plane contains more points, the weight of this plane is greater.

Localization precision in the proposed method is contingent solely upon camera accuracy. Provided that planes can be accurately matched, localization accuracy is influenced only by the camera or other visual systems’ precision. Plane matching correctness relies on map complexity and historical motion patterns, not camera accuracy–a notable advantage over Schaub *et al*. [36]. In instances where two planes exhibiting similar angles and distances occupy the field of view, identification errors might arise. However, real-world maps typically feature walls with perpendicular orientations and significant differences, making matching failures improbable. The a-priori information form employed in this method is succinct, necessitating only vertices to represent planes. This representation conserves storage and computational resources.

The proposed approach emphasizes real-time data processing for localization, achieving enhanced pose estimation results by leveraging historical data. Consequently, the indoor environment is intrinsically linked to the application of these methods, as flattened planes utilized for localization typically occur indoors.

This method’s characteristics encompass the need for initial position and orientation, and the mobile robot’s motion error must not be excessive. Should motion error between two localizations be too great, the input initial pose deviation for subsequent observation will be substantial, resulting in imprecise ROI calculation. Consequently, point cloud segmentation of the corresponding plane will be inaccurate, leading to localization errors. Ultimately, the tilt angles between localization planes within the scene should exhibit significant disparities to avert matching errors.

Our proposed method leverages prior information to segment partial point clouds from the field of view, which provides a significant advantage when dealing with some distorted point clouds in view. By focusing on the segmented relevant point clouds, the method remains unaffected by the distortion and maintains its localization effectiveness. This approach is particularly suitable for environments with complex or irregular geometries, where distortion in point cloud data is more likely to occur.

With the features talked above, the proposed method achieves robot localization in weak-feature and repetitive environments by capitalizing on prior plane information. This approach is well-suited for scenarios in which mobile robots adhere to predetermined paths during movement. If integrated with other localization methods, there is potential for realizing even more sophisticated and intelligent mobile robot localization.

## 6 Conclusion

This article introduces a mobile robot localization technique with a-priori planes. Initially, given the finite nature of planes in reality, they are represented by the vertices of planar closed shapes. All vertices in space are projected onto the proposed extended image coordinate system to ascertain whether the plane theoretically exists within the camera’s field of view. The theoretical visible vertices compose the filter ROI, which serves as a preliminary selection of points representing the target plane. Subsequently, the RANSAC method is employed to identify plane parameters. The filter ROI calculation is also contingent upon an initial known position and pose, which, however, need not be numerically precise.

Initially, the identified planes are represented in the camera coordinate system. A constraint equation system is established by integrating the plane equation in the camera coordinate system with the prior known plane in the ground coordinate system. This constraint equation system delineates the position and pose constraints for the mobile robot. When the constraint equation count exceeds two, weighted least square estimation is applied to determine the optimal position and pose.

Lastly, the redundancy of the initial known position and pose error is assessed based on the mobile robot prototype. Experimental results indicate that a certain degree of initial position and pose error does not impact plane identification outcomes. The proposed localization method is further validated during the pathway tracking experiment’s localization process. A high-precision motion capture system is employed to reflect the mobile robot vision system’s localization results.

Looking ahead, the integration of multi-source visual perception approaches will be further explored to achieve effective mobile robot localization in more complex scenarios.

## Supporting information

S1 Appendix Homogeneous transformation for planes. (PDF)

S1 Table Localization error in pathway tracking experiment. (PDF)
