# Supplementary material for: Localization on a-priori information of plane extraction
Source: PLoS One. 2023 May 8;18(5):e0285509. doi: 10.1371/journal.pone.0285509 (PMC10166544; doi:10.1371/journal.pone.0285509)
Supplement: S1 Appendix — (PDF) [file pone.0285509.s001.pdf]

## S1 Appendix. Homogeneous transformation for planes

Suppose that there is a plane, $\boldsymbol{\pi}$

$$\boldsymbol{\pi} = \left(\pi_1, \pi_2, \pi_3, \pi_4\right)^T \qquad \text{(A1)}$$

If there are the arbitrary homogenous coordinates $X = \left(x, y, z, 1\right)$ placed on the plane $\boldsymbol{\pi}$, then

$$\boldsymbol{\pi}^T X = 0 \qquad \text{(A2)}$$

As long as the point $X$ locates on the plane $\boldsymbol{\pi}$, Equation (A2) holds for any reference coordinate system.

Suppose that the homogenous transformation matrix $\boldsymbol{T}_{\mathrm{C}}^{\mathrm{R}}$ represents the transformation from the camera coordinate system and the robot coordinate system. Then,

$$X^R = \boldsymbol{T}_C^R X^C \qquad \text{(A3)}$$

In Equation (A2), $X^{\mathrm{R}}$ represents the homogenous coordinates of point $X$ under robot coordinate system. And $X^{\mathrm{C}}$ represents the homogenous coordinates of point $X$ under camera coordinate system.

According to Equation (A2), there is

$$\left(\boldsymbol{\pi}^{\mathrm{R}}\right)^{\mathrm{T}} X^{\mathrm{R}} = 0 \qquad \text{(A4)}$$

And

$$\left(\boldsymbol{\pi}^{\mathrm{C}}\right)^{\mathrm{T}} X^{\mathrm{C}} = 0 \qquad \text{(A5)}$$

Substitute Equation (A3) into Equation (A4) and (A5), there is

$$\left(\boldsymbol{\pi}^{\mathrm{C}}\right)^{\mathrm{T}} = \left(\boldsymbol{\pi}^{\mathrm{R}}\right)^{\mathrm{T}} \boldsymbol{T}_{\mathrm{C}}^{\mathrm{R}} \qquad \text{(A6)}$$

Take the transpose on both sides of Equation A6

$$\boldsymbol{\pi}^{\mathrm{C}} = \left(\boldsymbol{T}_{\mathrm{C}}^{\mathrm{R}}\right)^{\mathrm{T}} \boldsymbol{\pi}^{\mathrm{R}} \qquad \text{(A7)}$$

Since the homogeneous transformation matrix, $\boldsymbol{T}_{\mathrm{C}}^{\mathrm{R}}$ is an invertible matrix, hence

$$\boldsymbol{\pi}^{\mathrm{R}} = \left(\boldsymbol{T}_{\mathrm{C}}^{\mathrm{R}}\right)^{-\mathrm{T}} \boldsymbol{\pi}^{\mathrm{C}} \tag{A8}$$

The transformation equation of the homogenous plane can be expressed as Equation (A8). Similarly, the corresponding from the plane represented in the camera coordinate system to the plane represented in the robot coordinate system.

$$\boldsymbol{\pi}^{\mathrm{G}} = \left(\boldsymbol{T}_{\mathrm{R}}^{\mathrm{G}}\right)^{-\mathrm{T}} \left(\boldsymbol{T}_{\mathrm{C}}^{\mathrm{R}}\right)^{-\mathrm{T}} \boldsymbol{\pi}^{\mathrm{C}} \tag{A8}$$

Equation (A8) shows the transformation function for the homogenous plane that represents between two coordinate systems.
