# Supplementary material for: Localization on a-priori information of plane extraction
Source: PLoS One. 2023 May 8;18(5):e0285509. doi: 10.1371/journal.pone.0285509 (PMC10166544; doi:10.1371/journal.pone.0285509)
Supplement: S1 Table — (PDF) [file pone.0285509.s002.pdf]

S2 Table. Localization error in pathway tracking experiment

Motion seq.	Mechanical odometry (mm)	Proposed method (mm)
**1**	/	/
**2**	5.93	7.94
**3**	4.44	19.51
**4**	5.58	19.08
**5**	14.04	16.92
**6**	15.61	19.88
**7**	21.17	9.91
**8**	28.64	17.50
**9**	38.15	28.30
**10**	48.57	27.11
**11**	42.51	27.06
**12**	32.39	12.20
**13**	32.61	24.97
**14**	35.72	20.18
**15**	42.01	17.67
**16**	62.87	26.34
**17**	61.22	23.51
**18**	69.17	22.32
**19**	87.18	19.84
**20**	71.46	1.95
**21**	77.15	4.05
**22**	108.79	9.90
